# Human Tra2 proteins jointly control a *CHEK1* splicing switch among alternative and constitutive target exons

**DOI:** 10.1038/ncomms5760

**Published:** 2014-09-11

**Authors:** Andrew Best, Katherine James, Caroline Dalgliesh, Elaine Hong, Mahsa Kheirolahi-Kouhestani, Tomaz Curk, Yaobo Xu, Marina Danilenko, Rafiq Hussain, Bernard Keavney, Anil Wipat, Roscoe Klinck, Ian G. Cowell, Ka Cheong Lee, Caroline A. Austin, Julian P. Venables, Benoit Chabot, Mauro Santibanez Koref, Alison Tyson-Capper, David J. Elliott

**Affiliations:** 1 Institute of Genetic Medicine, Newcastle University, Central Parkway, Newcastle NE1 3BZ, UK; 2 School of Computing Science, Claremont Tower, Newcastle University, Newcastle upon Tyne NE1 7RU, UK; 3 Institute for Cellular Medicine, Newcastle University, Framlington Place, Newcastle NE2 4HH, UK; 4 Faculty of Computer and Information Science, University of Ljubljana, Trzaska cesta 25, SI-1000, Ljubljana, Slovenia; 5 Institute of Cardiovascular Sciences, The University of Manchester, Manchester M13 9NT, UK; 6 Department of Microbiology and Infectious Diseases, Faculty of Medicine and Health Sciences, Université de Sherbrooke, Sherbrooke, Québec, Canada J1E 4K8; 7 Institute for Cell and Molecular Biosciences, Newcastle University, Newcastle NE2 4HH, UK

## Abstract

Alternative splicing—the production of multiple messenger RNA isoforms from a single gene—is regulated in part by RNA binding proteins. While the RBPs transformer2 alpha (Tra2α) and Tra2β have both been implicated in the regulation of alternative splicing, their relative contributions to this process are not well understood. Here we find simultaneous—but not individual—depletion of Tra2α and Tra2β induces substantial shifts in splicing of endogenous Tra2β target exons, and that both constitutive and alternative target exons are under dual Tra2α–Tra2β control. Target exons are enriched in genes associated with chromosome biology including *CHEK1*, which encodes a key DNA damage response protein. Dual Tra2 protein depletion reduces expression of full-length CHK1 protein, results in the accumulation of the DNA damage marker γH2AX and decreased cell viability. We conclude Tra2 proteins jointly control constitutive and alternative splicing patterns via paralog compensation to control pathways essential to the maintenance of cell viability.

## Abstract (web summary)

RNA binding proteins are key regulators of alternative splicing. Here, Best *et al*. show that the human Tra2α and Tra2ß RNA binding proteins jointly contribute to the control of constitutive and alternative splicing events to regulate essential biological processes including the response to DNA damage.

## Graphical abstract

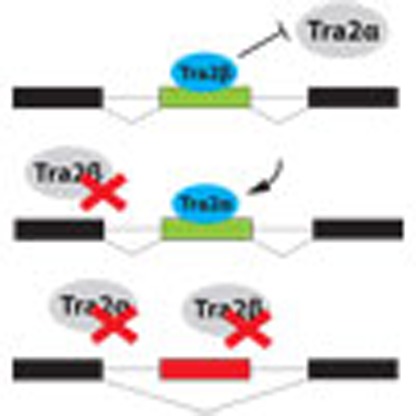

Human genes encode long precursor messenger RNAs (mRNAs) that are extensively processed before nuclear export. This maturation includes the splicing of exons, which normally occurs with high fidelity to create functional mRNAs^1^. Constitutive exons splice into all mRNAs transcribed from a gene, while alternative exons are sometimes included and sometimes skipped^2^. Human protein-coding genes each produce an average of three mRNA isoforms through alternative splicing, many of which are differentially regulated^3^. RNA binding proteins play a key role in transforming precursor RNAs into mRNAs. Although RNA-binding proteins can regulate many transcripts in parallel, some splicing regulatory proteins preferentially engage with transcripts belonging to specific functional classes, including Nova proteins (synapse functions), Fox proteins (neuromuscular, cytoskeleton and EMT functions), PTB proteins (cytoskeleton functions) and T-STAR (synapse functions)^4^,^5^,^6^,^7^,^8^.

Transformer2 (Tra2) proteins are involved in splicing control^9^,^10^. First discovered in insects, Tra2 proteins form an essential component of the alternative splicing complex that controls fly sexual differentiation^11^,^12^. Tra2 proteins are conserved across the animal kingdom, but separate gene paralogs encoding Tra2α and Tra2β proteins evolved early in vertebrate evolution^13^,^14^. Knockout experiments in mice show that Tra2β is essential for embryonic and brain development^15^,^16^,^17^,^18^. In humans, Tra2β expression levels change in several cancers (reviewed by Best *et al*.^19^), and Tra2β is implicated in the pathology of other diseases including spinal muscular atrophy^20^, Alzheimer’s disease^21^ and frontotemporal dementia and Parkinsonism linked to chromosome 17 (ref. 22).

Tra2 proteins have amino- and carboxy-terminal domains enriched in arginine and serine residues (RS domains) flanking a single central RNA recognition motif (RRM) and so resemble the relatively well characterized core group of 12 SR proteins that control both constitutive and alternative splicing as well as other aspects of RNA metabolism^23^,^24^,^25^. Each core SR protein contains N-terminal RRMs and single C-terminal RS domains. However, unlike the core SR proteins all current data implicate Tra2 proteins solely in alternative splicing rather than constitutive splicing^10^,^26^, and only SR proteins and not Tra2 proteins can provide splicing activity to S100 extracts^26^.

To regulate splicing inclusion Tra2β binds to AGAA-rich and CAA-rich target RNA sequences. These RNA protein interactions have been resolved at the atomic level^9^,^27^. Endogenous Tra2β target RNAs have been identified using HITS-CLIP^18^, RIP-seq^28^, shRNA depletion^29^ and microarrays^17^, but important fundamental questions still remain as to the identity of the biological targets and the functions of vertebrate Tra2 proteins. These include whether endogenous Tra2α and Tra2β proteins jointly control the same splicing targets, and if so what these shared targets are? Although Tra2α and Tra2β both activate splicing of the same model exons when overexpressed in transfected HEK-293 cells (suggesting redundant functions)^18^, the *Tra2a* gene alone is not sufficient to maintain viability in *Tra2b* knockout mice (suggesting specific functions)^15^. Another question relates to how Tra2α and Tra2β interact with each other? We previously found that Tra2β protein binds to a poison exon in the *TRA2A* gene to activate poison exon inclusion^18^. Poison exons introduce premature translation termination codons into mRNAs so as to inhibit translation of full-length proteins and are often regulatory^18^,^30^,^31^,^32^, but whether Tra2α might reciprocally control Tra2β expression is not known.

Here we address these questions in human MDA-MB-231 cells that model invasive breast cancer. We find asymmetric splicing feedback control pathways between Tra2α and Tra2β that buffer splicing defects caused by depletion of either Tra2α or Tra2β protein alone. Overriding these feedback control pathways by joint depletion of both Tra2α and Tra2β globally identifies Tra2-dependent target exons, and reveals critical roles for these proteins in DNA damage control and cell viability.

## Results

### Tra2β efficiently suppresses Tra2α protein expression

**Figure 1:**
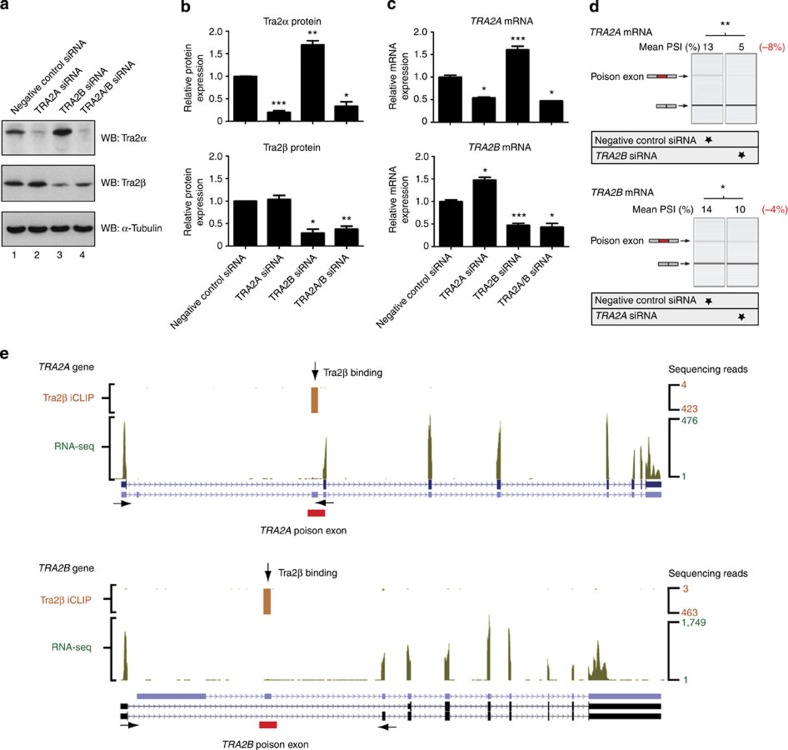
Tra2β regulates Tra2α protein expression. (**a**) Western blot analysis showing depletion of *TRA2B* induces reciprocal upregulation of Tra2α protein expression, whereas depletion of *TRA2A* had minimal effect on Tra2β protein expression. (**b**) Quantitation of cross-regulation between Tra2α and Tra2β at the protein level (Tra2α and Tra2β protein expression were quantified relative to α–Tubulin from three western blots using independent biological replicates). (**c**) Quantitation of cross-regulation between Tra2α and Tra2β at the RNA level from quantitative PCR analysis of three independent biological replicates in MDA-MB-231 cells. (**d**) Splicing inclusion of the *TRA2A* poison exon is strongly reduced by depletion of endogenous Tra2β protein, whereas splicing inclusion of the *TRA2B* poison exon is less affected by depletion of Tra2α protein. Splicing patterns were monitored by RT–PCR between flanking exons (arrowed) followed by capillary electrophoresis. (**e**) Screenshot from the UCSC genome browser^35^ showing the *TRA2B* and *TRA2A* genes, and the positions of aligned RNA-seq reads (green peaks) and Tra2β binding (orange clusters of significant cross-linking by Tra2β protein identified by biological triplicate iCLIP experiments) in MDA-MB-231 cells. Probability (*P*) values were calculated using an independent two-sample *t*-test between negative control siRNA-treated cells and the gene-specific siRNA-treated cells (statistical significance shown as: *P<0.05, **P<0.01, ***P<0.0001). All data represented by bar charts was generated from three biological replicates and error bars represent the s.e.m.

To test for *in vivo* interactions between Tra2α and Tra2β proteins, we monitored their expression levels using western blots. Consistent with predictions from our previous study^18^, Tra2α protein levels were normally very low but significantly increased after small interfering RNA (siRNA)-mediated depletion of Tra2β (Fig. 1a top panel, compare lanes 1 and 3, and Fig. 1b). Although weak, the Tra2α western blot signal was of the predicted size and was almost completely eliminated following transfection with a *TRA2A*-specific siRNA (Fig. 1a top panel, compare lanes 1 and 2). Tra2α protein depletion had less effect on Tra2β protein levels (Fig. 1a, middle panel and Fig. 1b). Western blot analysis confirmed this effect for two independent sets of siRNAs targeted against different parts of the respective mRNAs (Supplementary Fig. 1).

Consistent with Tra2β protein repressing Tra2α expression via poison exon activation, siRNA-mediated depletion of Tra2β led to strongly reduced splicing inclusion of the *TRA2A* poison exon (Fig. 1d, upper panel). siRNA-mediated depletion of Tra2α protein led to a smaller but detectable effect on splicing inclusion of the *TRA2B* poison exon (Fig. 1d, lower panel). Analysis of *TRA2A* and *TRA2B* steady state mRNA expression levels by quantitative PCR confirmed that each protein also negatively regulates the expression of the other at the RNA level (Fig. 1c).

### The *TRA2A* and *TRA2B* genes are differentially expressed

RNA-seq of MDA-MB-231 cells indicated that the *TRA2B* gene is expressed at much higher levels than the *TRA2A* gene (Fig. 1e shows one of three biological replicate RNA-seq analyses, with the height of the *y* axis showing read depth and so indicating relative gene expression levels). This provides a potential mechanism for why Tra2β represses Tra2α protein expression more than vice versa, since lower cellular concentrations of Tra2α would be less able to activate splicing of the *TRA2B* poison exon.

We used iCLIP^33^ to systematically map the transcriptome-wide binding sites of human Tra2β in MDA-MB-231 cells. Endogenous Tra2β protein was efficiently immunoprecipitated along with radiolabelled crosslinked RNA. A single radiolabelled RNA protein adduct of ~40 kDa was identified at high RNase concentrations, just above the known molecular weight of uncrosslinked endogenous Tra2β protein (37 kDa) (arrowed in Supplementary Fig. 2a). Lower RNase concentrations enabled endogenous Tra2β binding sites to be mapped across the MDA-MB-231 cell transcriptome in biological triplicate iCLIP experiments. Following deep sequencing, 7,443,903 reads were successfully mapped back to the human genome, of which 3,338,710 were unique cDNA reads used for downstream analysis (Supplementary Data 1). These individual sequencing reads are subsequently referred to as iCLIP tags. The only clusters of Tra2β iCLIP tags, which mapped to the human *TRA2B* and *TRA2A* genes from all three biological replicates were within their respective poison exons (Fig. 1e). Despite much lower levels of overall *TRA2A* gene expression, the *TRA2A* poison exon had a similar number of Tra2β iCLIP tags as the *TRA2B* poison exon. This suggests the *TRA2A* poison exon is a stronger physiological target for Tra2β binding than the *TRA2B* poison exon (the *TRA2A* poison exon also has a much higher density of AGAA Tra2β binding sites than the *TRA2B* poison exon^18^).

### Endogenous Tra2α functionally compensates for loss of Tra2β

The most frequently enriched pentamers recovered in the iCLIP tags were highly enriched in AGAA nucleotide sequences (Supplementary Fig. 2b), which is the Tra2β binding site predicted by HITS-CLIP for endogenous mouse Tra2β, RIP-seq and from SELEX experiments using purified Tra2β proteins^18^,^26^,^28^. However, our combined human iCLIP data in MDA-MB-231 cells provided substantially more coverage than previously obtained in mouse testis^18^ (in which just 177,457 reads were mapped back to the mouse genome). In total, 1,546,290 (44.8%) of unique cDNAs mapped to intronic regions, suggesting the Tra2β iCLIP experiment largely captured Tra2β interactions with pre-mRNAs. However, a further 1,169,374 (33.8%) of unique cDNAs mapped to exons (5′UTR, 3′UTR or ORF), despite exons comprising only ~1% of the genome. After correcting for the relative size of each genomic region (by dividing the number of unique cDNAs mapping to each genomic region by the relative size of that region within the genome), we find that Tra2β binding is highly enriched within exons: 76.8% of Tra2β iCLIP tags mapped to exons (5′UTR, 3′UTR or ORF), while a further 20.4% mapped to non-coding RNAs (Supplementary Fig. 2c).

We used our iCLIP data to screen for endogenous exons controlled by human Tra2β. Alternative exon junctions (+/−300 bp of either splice site) were stratified to find those with the highest number of iCLIP tags relative to overall iCLIP coverage within the same gene. To test whether upregulation of Tra2α protein expression could functionally compensate in splicing regulation for depletion of Tra2β, we then monitored percentage splicing inclusion (PSI) of associated exons after single depletion of either Tra2α or Tra2β, or after combined depletion of both Tra2α and Tra2β proteins.

**Figure 2:**
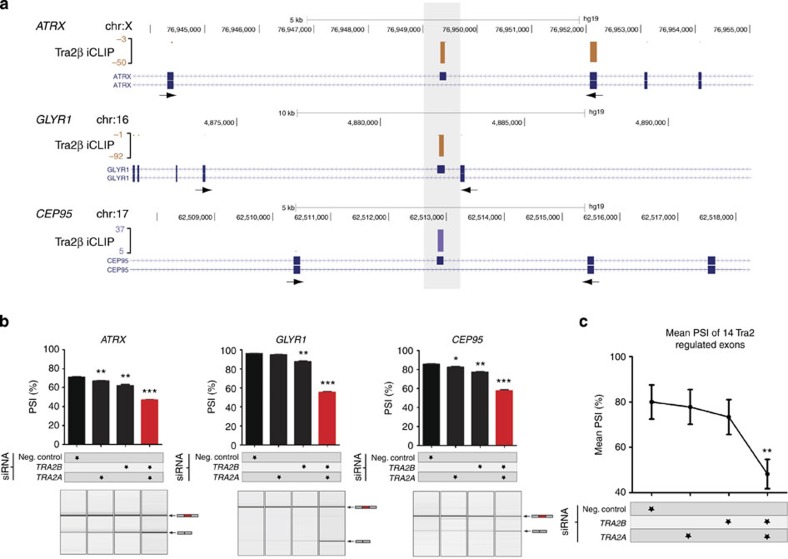
Endogenous Tra2α functionally compensates for loss of Tra2β. (**a**) UCSC genome browser screenshot^35^ showing significant clusters of iCLIP tags mapping directly to alternatively spliced exons within the *CEP95*, *GLYR1* and *ATRX* genes (position of target alternative exons highlighted in grey). (**b**) Splicing inclusion of novel Tra2β target exons within *ATRX*, *GLYR1* and *CEP95* were only slightly affected by depletion of either endogenous Tra2α or Tra2β proteins, but were strongly affected by joint depletion of both Tra2α and Tra2β (red). PSI levels were measured by RT–PCR and capillary gel electrophoresis (lower panels) in three biological replicates (upper panels). (**c**) Splicing inclusion of 14 novel Tra2β target exons showed minimal splicing response to single depletion of either Tra2α or Tra2β, but showed highly significant splicing changes after joint depletion of both Tra2 proteins (complete data for all 14 exons is provided in Supplementary Fig. 4). Probability (*P*) values were calculated using an independent two-sample *t*-test between PSI levels of negative control siRNA-treated cells and *TRA2A*/*TRA2B* siRNA-treated cells (statistical significance: *P<0.05, **P<0.01, ***P<0.0001). All data represented by bar charts was generated from three biological replicates where error bars represent the s.e.m.

Clusters of Tra2β iCLIP tags mapped to alternative exons in the *ATRX*, *GLYR1* and *CEP95* genes (Fig. 2a). Single depletion of either Tra2α or Tra2β had only a small effect on the endogenous splicing pattern of these three exons, but joint depletion of both Tra2α and Tra2β substantially decreased their splicing inclusion (Fig. 2b). We obtained similar results with 14/14 Tra2β target exons identified by iCLIP analysis (Fig. 2c. Individual data for each of these tested exons are shown in Supplementary Fig. 3). In fact, of these 14 tested exons, some less-responsive exons including cassette exons within *PAM* and *BDP1* only responded to depletion of both Tra2 proteins, and not to single depletion of Tra2β at all (Supplementary Fig. 3).

These data are consistent with maintenance of splicing patterns via paralog compensation, that is, following depletion of Tra2β, upregulated Tra2α is able to functionally substitute for Tra2β and largely maintain Tra2 target exon inclusion. The Tra2β target exons inhibited more substantially by joint Tra2 protein depletion compared with single depletion of either Tra2α or Tra2β included *SMN2* exon 7 (Supplementary Fig. 3), which is a candidate target for gene therapy in spinal muscular atrophy^20^.

### Tra2α and Tra2β control constitutive exon splicing patterns

**Figure 3:**
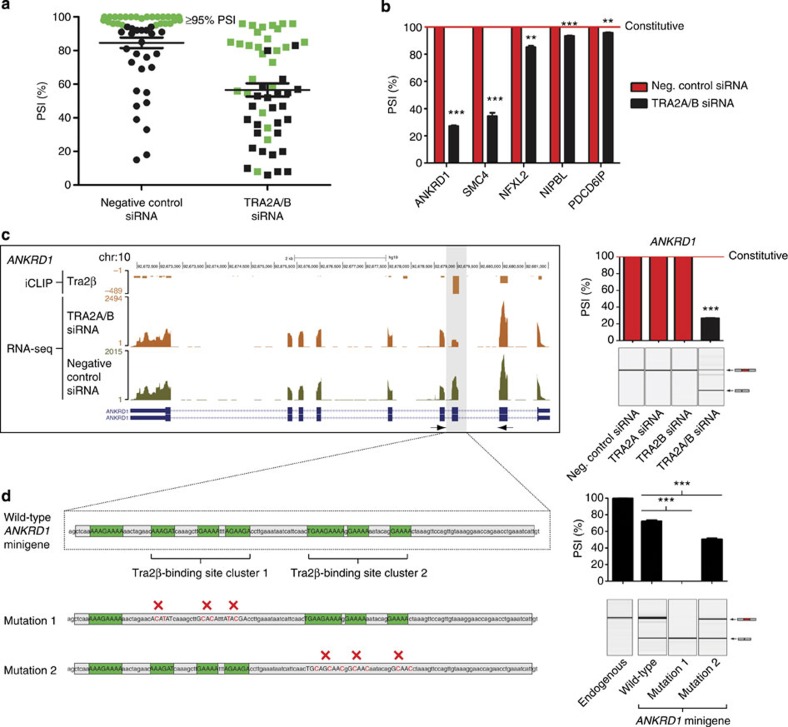
Tra2 proteins control splicing of constitutively spliced target exons. (**a**) Many novel Tra2α/β-responsive exons normally have high levels of splicing inclusion in MDA-MB-231 cells. PSI levels of target exons are shown from negative control siRNA-treated cells and after joint Tra2 protein depletion. Exons included at equal to or greater than 95% PSI in control MDA-MB-231 cells are highlighted in green. (**b**) The inclusion of five constitutively spliced Tra2β target exons is reduced after joint Tra2α/Tra2β protein depletion. PSI levels were measured by RT–PCR and capillary gel electrophoresis. (**c**) The splicing profile of the *ANKRD1* gene changes in response to joint depletion of Tra2 proteins. Combined iCLIP and RNA-seq data were visualized on the UCSC genome browser^35^ (left panel), and splicing inclusion levels directly measured using RT–PCR (right panel). Probability (*P*) values were calculated using an independent two-sample *t*-test between the PSI levels of negative control siRNA-treated cells and the gene-specific siRNA-treated cells (statistical significance: *P<0.05, **P<0.01, ***P<0.0001). All data represented by bar charts was generated from three biological replicates and error bars represent s.e.m. (**d**) Tra2β binding sites in the *ANKRD1* exon are essential for its splicing inclusion. The wild-type minigene contained two clusters of Tra2β binding sites, which were independently altered by mutagenesis (left panel). The PSI of the resulting exons was measured after transfection into HEK-293 cells at endogenous Tra2β protein concentrations (right panel). Probability (*P*) values were calculated using an independent two-sample *t*-test between PSI levels of the wild-type and mutated versions of the *ANKRD1* minigene (statistical significance: *P<0.05, **P<0.01, ***P<0.0001). All data represented by bar charts was generated from three biological replicates and error bars represent the s.e.m.

Splicing profiles of candidate Tra2β target exons (containing Tra2β iCLIP tag coverage) were next analysed using RNA-seq after joint depletion of Tra2α and Tra2β proteins, and changes validated by reverse transcriptase (reverse transcriptase–PCR). From the initial panel of 30 Tra2 protein-responsive alternative exons that we identified, 7/30 were included at 100% and 17/30 had a PSI greater or equal to 95% in MDA-MB-231 cells (Fig. 3a). These results suggested that Tra2 proteins might be important for the inclusion of constitutive exons (in addition to their expected function in alternative exon splicing regulation), or alternatively that Tra2 protein expression levels in MDA-MB-231 cells are sufficient to promote 100% inclusion of some alternative exons.

To distinguish between these possibilities, we used our iCLIP and RNA-seq data to search for splicing changes in exons that have never previously been annotated as alternatively spliced in human cells^34^,^35^. Such exons were found in the *ANKRD1*, *SMC4*, *NFXL2*, *NIPBL* and *PDCD6IP* genes. Each of these five exons were spliced at 100% PSI in MDA-MB-231 cells but skipped at different levels after Tra2 protein depletion (Fig. 3b). The constitutively spliced exon within *ANKRD1* showed the largest change (Fig. 3c), a -73% point switch in PSI after joint depletion of Tra2α and Tra2β. To further dissect splicing control, we cloned the *ANKRD1* exon and its flanking intronic sequences between β–globin exons in a minigene construct. Transfection experiments showed the *ANKRD1* exon is included at 72% PSI when expressed from this minigene, indicating it lacks some important sequences for splicing (the endogenous *ANKRD1* exon was 100% included in these transfected HEK-293 cells, Fig. 3d). Two clusters of GAA-rich Tra2β binding sites were present in the *ANKRD1* exon (Tra2β binding site clusters 1 and 2). Mutation of either Tra2β binding site cluster negatively impacted splicing inclusion, showing an essential role for these binding sites in modulating the inclusion of the *ANKRD1* exon. In particular, mutation of Tra2β binding site cluster 1 (to create Mutant M1) completely abolished splicing inclusion of the *ANKRD1* constitutive exon (Fig. 3d, right panel).

**Figure 4:**
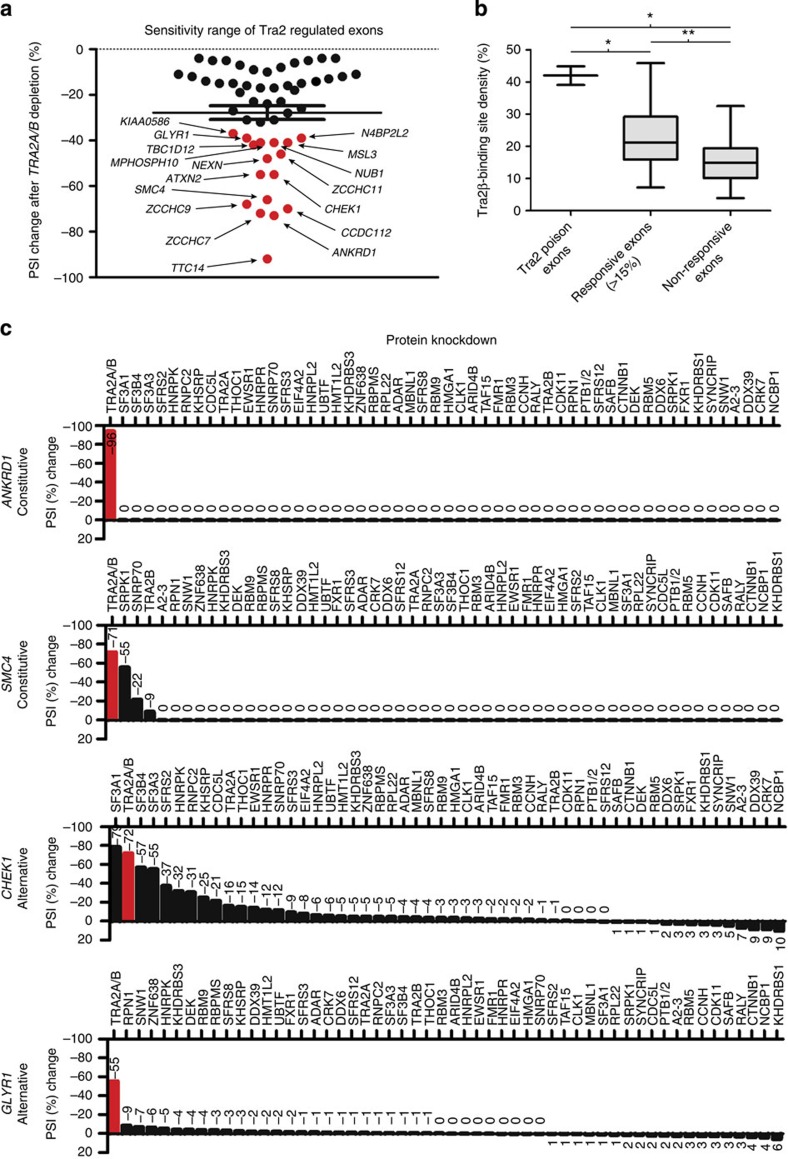
Identification of exons jointly controlled by Tra2α and Tra2β proteins. (**a**) Scatterplot showing amplitude of splicing response of 53 exons to joint depletion of endogenous Tra2α and Tra2β in MDA-MB-231 cells. The genes corresponding to the highest amplitude PSI changes after joint Tra2 protein depletion are labelled and highlighted in red. (**b**) Analysis of Tra2β binding site density (measured as a percentage of exon content) within groups of Tra2β target exons identified by iCLIP. Tra2β binding site density comparisons are shown between the Tra2α and Tra2β poison exons; all exons that showed a greater than 15% point PSI change following joint Tra2α and Tra2β depletion; and in the exons that bound Tra2β based on iCLIP tag coverage but did not respond to Tra2α and Tra2β depletion. Probability (*P*) values were calculated using an independent two-sample *t*-test (statistical significance: *P<0.05, **P<0.01, ***P<0.0001). (**c**) Regulation of *ANKRD1*, *SMC4*, *GLYR1* and *CHEK1* splice variants following knockdown of a panel of RNA binding proteins (RBPs) in MDA-MB-231 cells^5^. The *y* axis shows PSI change after joint Tra2α and Tra2β depletion, with a negative number indicating splicing repression in the absence of these proteins.

In total, we identified and validated 53 human splicing targets, which were both directly bound and jointly controlled by Tra2 proteins, including both alternative and constitutive exons (Fig. 4a; Supplementary Fig. 10; Supplementary Data 2). As well as *SMN2* exon 7 (ref. 20), these included the *NASP-T* exon and the *TRA2A* poison exon, orthologs of which have both been previously identified as functional Tra2β splicing targets in the mouse testis by HITS-CLIP^18^. The *NASP-T* exon was also in the data set of Tra2β targets identified by RIP-seq^28^. However, the vast majority of the dual Tra2α/Tra2β target exons identified here are novel. The PSI changes for individual genes in response to joint Tra2α and Tra2β protein depletion ranged between −4 and −92% points (measured by RT–PCR, Fig. 4a; Supplementary Data 2), indicating that individual Tra2-dependent exons have different intrinsic requirements for Tra2 proteins. The length of Tra2-dependent exons ranged from 64 nucleotides at the smallest (a cassette exon in the *SMYD2* gene, which showed a −5% point switch in PSI in response to Tra2 protein depletion), to 5916 nucleotides at the largest (an unusually large internal cassette exon in the *SON* gene, which showed a −8 point PSI change in response to endogenous Tra2 protein depletion) (Supplementary Data 2). A further 38 exons had Tra2β iCLIP tag coverage, but did not detectably respond to Tra2 protein depletion in MDA-MB-231 cells: possibly this latter class of exons either might need a relatively small amount of Tra2 protein to be included, or alternatively they might not be controlled by Tra2 proteins in MDA-MB-231 cells (Supplementary Data 2). In a comparison between Tra2 responsive and non-responsive exons, the only statistically significant difference was the density of Tra2β binding sites in the more highly responsive exons (exons showing >15 PSI change following joint Tra2α/Tra2β depletion, Fig. 4b). High resolution iCLIP maps of the individual exons are shown in Supplementary Fig. 10.

### Tra2 proteins are highly specific splicing regulators

To establish the relative role of Tra2 proteins in controlling the identified panel of target exons, we probed a custom plate containing cDNAs, where we had systematically knocked down 53 known splicing regulators in MDA-MB-231 cells^5^ (Fig. 4c). Strikingly, of all the knockdowns tested, only double knockdown of Tra2 proteins shifted *ANKRD1* splicing (constitutive exon) and joint Tra2 depletion also had the largest effect on splicing for *GLYR1* (alternative exon). An intermediate situation was observed for *SMC4* (constitutive exon) in which knockdown of *SNRP70* (encoding U170K) also reduced splicing inclusion, as did knockdown of *SRPK1*. The splicing inclusion pattern of *CHEK1* was strongly shifted (−78 point PSI switch) by joint depletion of Tra2 proteins, but consistent with broader mechanisms of combinatorial control, significant shifts were also seen after depletion of three core U2 snRNP components, which are thought to be important for splice site commitment for all exons. Depletion of other constitutive splicing factors such as SFRS2, hnRNPK, hnRNPC2, KHSRP and CDC5L also affected *CHEK1* exon 3 splicing.

Since our panel of splicing factor knockdowns was not exhaustive, we cannot exclude all combinations of combinatorial control. However, our data are at least consistent with Tra2 proteins being among the most quantitatively important splicing regulators for their individual target exons.

### Tra2 splicing targets associate with chromosome biology

**Figure 5:**
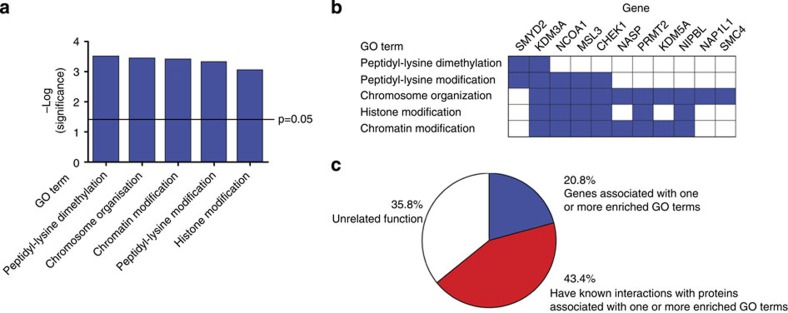
Tra2 splicing targets are enriched in GO terms associated with chromosome biology. (**a**) GO enrichment analysis reveals splicing targets responsive to endogenous Tra2α/Tra2β protein concentrations are enriched in particular biological processes associated with chromosome biology. (**b**) GO enrichment analysis showed some joint Tra2α/Tra2β-responsive exons were annotated to multiple overlapping biological processes. (**c**) Summary of GO and network analyses of joint Tra2α and Tra2β-dependent splicing targets. Individual segments of the pie chart show the percentage of Tra2α/Tra2β target genes directly annotated to the GO biological processes shown in part **a**; the percentage of Tra2α/Tra2β target genes that interact within the BioGrid database with partners known to be involved in the biological processes shown in part **a**; and the percentage of Tra2α/Tra2β target genes, which have unknown or unrelated functions. Full details of the BioGrid analysis are given in Supplementary Fig. 4.

Gene ontology (GO) enrichment analysis of the 53 human genes containing Tra2-dependent exons revealed an enrichment of five functionally similar biological processes (Fig. 5a). Eleven of the 53 genes were annotated to one or more of these processes, with ten of the eleven genes being annotated to the term ‘chromosome organization’. There was significant overlap in annotation between this process and annotation to the conceptually related terms ‘histone modification’ and ‘chromatin modification’ (Fig. 5b).

The BioGRID database^36^ was used to retrieve a network of functional interaction data involving genes containing Tra2-dependent exons. In addition to the eleven genes directly annotated to the five enriched GO processes in Fig. 5a, a further 23 of the 53 genes (43.4%) that contain Tra2-dependent exons also have functional interactions with genes annotated to these terms (Supplementary Fig. 4, and summarized in Fig. 5c). Interestingly, although indirectly connected within the network via these annotated genes, none of the 53 genes containing validated Tra2-dependent exons directly interact with one another in the BioGRID database (Supplementary Fig. 4).

### Tra2 proteins control splicing of a key checkpoint protein

**Figure 6:**
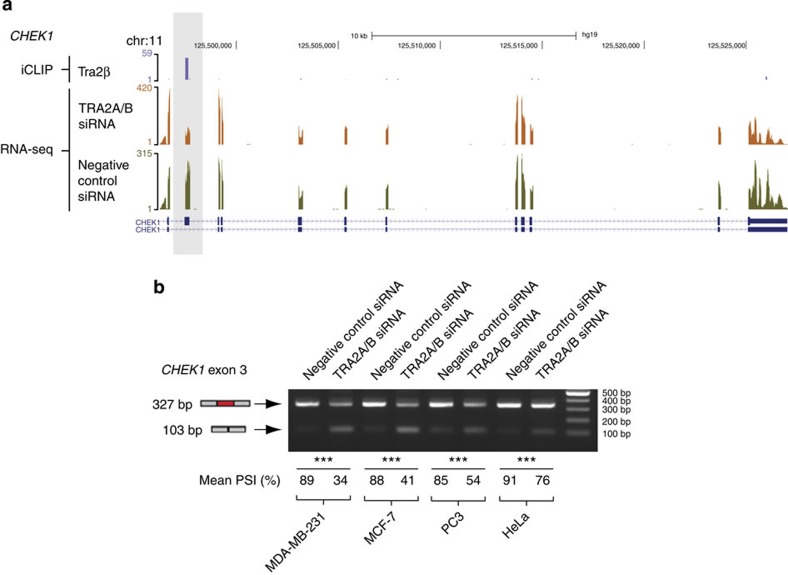
**Splicing inclusion of** ***CHEK1*** **exon 3 decreases following joint depletion of Tra2α and Tra2β.** (**a**) Screenshot^35^ from the UCSC genome browser showing the *CHEK1* gene. Tra2β iCLIP tags are shown at the top in purple, and map predominantly to a known alternative exon (*CHEK1* exon 3) that is skipped in one mRNA isoform (shaded grey). Example RNA-seq tracks from control MDA-MB-231 cells and after joint Tra2α and Tra2β protein depletion are shown in green and orange respectively. (**b**) Splicing inclusion of *CHEK1* exon 3 is inhibited by siRNA-mediated Tra2α and Tra2β protein depletion in multiple cell types. Splicing inclusion was monitored by RT–PCR and agarose gel electrophoresis, and shown as a mean PSI value after analysis of three independent samples. Probability (*P*) values were calculated using an independent two-sample *t*-test between PSI levels of negative control siRNA-treated cells and TRA2A/B siRNA-treated cells (statistical significance: *P<0.05, **P<0.01, ***P<0.0001). An uncropped gel is shown in Supplementary Fig. 13.

Among the Tra2 target exons involved in chromosome biology was exon 3 of the *CHEK1* gene, which encodes the serine/threonine protein kinase CHK1 that is involved in checkpoint control in response to DNA damage. iCLIP analysis identified significant Tra2β binding over *CHEK1* exon 3 (Fig. 6a), and we observed a −55 point PSI switch for this exon in MDA-MB-231 cells after joint Tra2 protein depletion (Fig. 6b). Joint depletions of Tra2α and Tra2β proteins also indicate *CHEK1* exon 3 splicing is under similar control in multiple cell types including MCF7, PC3 and HeLa (Fig. 6b).

**Figure 7:**
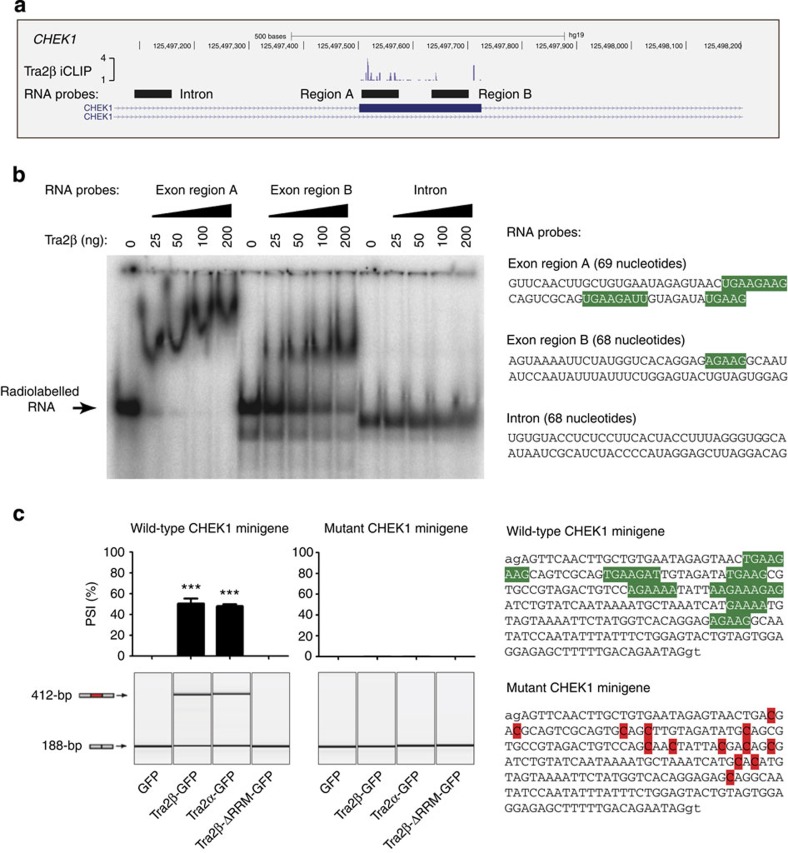
**Human Tra2α and Tra2β regulate** ***CHEK1*** **exon 3 through direct RNA protein interactions.** (**a**) High resolution map *CHEK1* exon 3 showing mapped iCLIP tags (purple bars) and the three subregions of the pre-mRNA, which were used to generate RNA probes for EMSAs. This screenshot was downloaded from the UCSC genome browser^35^. (**b**) Molecular interactions between purified Tra2β protein and RNA probes in and around *CHEK1* exon 3 (the location of these probes is shown in part **a**). The sequences of the probes are shown to the right, with predicted Tra2β binding sites shaded green. (**c**) Splicing patterns of mRNAs made from a minigene containing *CHEK1* exon 3 in response to coexpressed fusion proteins, expressed either as a PSI (upper bar chart, *n*=3 independent experiments) or shown as one of the original capillary electrophoresis gel-like images from a single experiment (lower image). The sequence of *CHEK1* exon 3 is shown to the right, with the predicted Tra2β binding sites shaded green (above) and the altered sequence after these sites were mutated (below, the altered nucleotides are shaded red). Probability (*P*) values were calculated using an independent two-sample *t*-test between PSI levels of the minigene-derived CHEK1 exon 3 in cells cotransfected with GFP and each of the different Tra2 constructs (statistical significance: *P<0.05, **P<0.01, ***P<0.0001). All data represented by bar charts was generated from three biological replicates and error bars represent s.e.m.

Tra2β iCLIP tags mapped throughout *CHEK1* exon 3, but were particularly enriched towards the 3′ splice site (Fig. 7a). We confirmed *CHEK1* exon 3 is a direct target for Tra2β binding *in vitro* by electrophoretic mobility shift assays (EMSAs) using radiolabelled RNA probes corresponding to portions of the exon sequence (Fig. 7b). RNA probe A corresponds to the part of *CHEK1* exon 3 with the most Tra2β iCLIP tags, and also contains the most predicted binding sites for Tra2β (shaded green in Fig. 7B, right hand side). RNA probe A was very efficiently shifted by even the lowest tested concentrations (25 ng) of Tra2β protein. RNA probe B did not bind Tra2β protein as tightly (around 200 ng Tra2β protein was needed to see a comparable shift) and also contained fewer Tra2β binding sites and mapped iCLIP tags. A control RNA probe corresponding to the flanking intron sequence did not shift even at the highest concentrations of Tra2β protein (this intron sequence contained no predicted Tra2β binding sites).

We confirmed that *CHEK1* exon 3 is a direct target for Tra2β splicing regulation using a minigene construct in which *CHEK1* exon 3 is flanked by β-globin exons (Fig. 7c). After transfection of this minigene into HEK-293 cells, *CHEK1* exon 3 was skipped, but its splicing inclusion was strongly induced in response to co-transfection with either Tra2β-GFP or Tra2α-GFP. No *CHEK1* exon 3 splicing activation was observed after co-transfection of either Tra2βΔRRM-GFP (lacking the RRM) or GFP alone. Furthermore, point mutations of the Tra2β binding sites within the exon (wild-type binding sites shaded green, mutations shaded red in Fig. 7c right hand side) completely abolished splicing activation in response to coexpressed Tra2 proteins.

### Tra2 proteins are required for CHK1 protein expression

**Figure 8:**
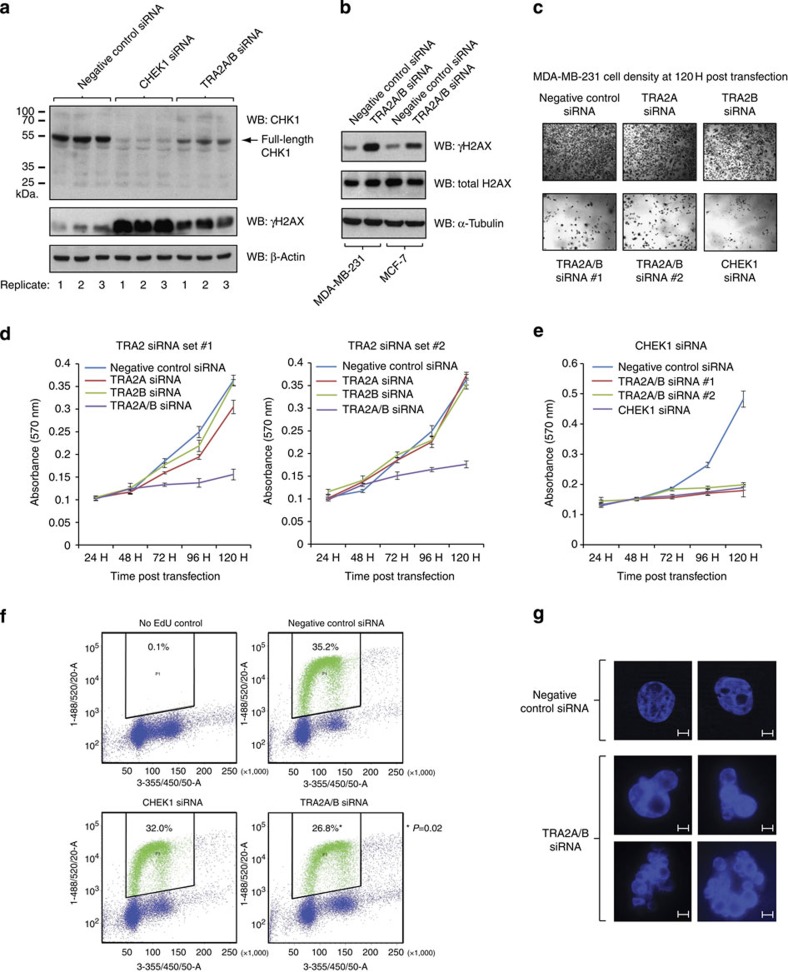
Human Tra2 proteins are essential for expression of full-length CHK1 protein and to maintain cell viability. (**a**) Full-length CHK1 protein expression is depleted by siRNAs specific to *CHEK1* mRNA and also by joint siRNAs specific to the *TRA2A* and *TRA2B* mRNAs. In each case, samples from three independent replicate experiments were analysed in parallel. Also detected in these samples are levels of γH2AX and α-tubulin. (**b**) Expression of total H2AX and γH2AX after joint Tra2α and Tra2β depletion, or depletion with a control siRNA in MDA-MB-231 cells and MCF7 cells. (**c**) Measurement of cell density 120 h after transfection of siRNAs targeting different regions of the *TRA2A* and *TRA2B* mRNAs or *CHEK1* mRNA. (**d**) Joint (but not single) depletion of Tra2α and Tra2β proteins reduced MDA-MB-231 cell viability measured by MTT assays after siRNA transfection. (**e**) Depletion of CHK1 protein alone was sufficient to reduce viability of MDA-MB-231 cells measured by MTT assay at different time points after siRNA transfection. (**f**) Joint depletion of Tra2α and Tra2β reduced the proportion of EdU-positive MDA-MB-231 cells 96 h after siRNA transfection. Separate panels, shown clockwise from top left, show fluorescence-activated cell sorting analysis of control MDA-MB-231 cells incubated without EdU; cells transfected with a negative control siRNA and incubated with EdU; cells transfected with siRNAs specific for *TRA2A* and *TRA2B* and incubated with EdU; and cells transfected with a single siRNA specific to *CHEK1* and incubated with EdU. Probability (*P*) values were calculated using an independent two-sample *t*-test comparing the percentage of EdU-positive cells of negative control siRNA-treated cells and the gene-specific siRNA-treated cells (statistical significance: *P<0.05, **P<0.01, ***P<0.0001). Data were generated from three biological replicates. (**g**) Examples of abnormal nuclear morphology observed within cells transfected with siRNAs specific for *TRA2A* and *TRA2B* (lower panel) compared with the normal morphology seen in negative control siRNA-treated cells (upper panel). Cells were stained with 4',6-diamidino-2-phenylindole, and these images were taken 96 h after siRNA transfection. The scale bar shows 5 μM. Uncropped western blots are shown in Supplementary Figs 11–15.

We carried out further experiments to test if Tra2 proteins are also required for expression of full-length CHK1 protein. On western blots, we could detect expression of a single major protein CHK1 isoform in MDA-MB-231 cells, corresponding to the expected size of full-length CHK1 protein (54 kDa). This band was substantially reduced following treatment with an siRNA directed against *CHEK1* mRNA (Fig. 8a). Consistent with joint control of *CHEK1* expression by Tra2α and Tra2β, levels of full-length CHK1 protein were also substantially reduced after joint depletion of Tra2α and Tra2β. Expression of full-length CHK1 protein was also reduced after joint Tra2α and Tra2β protein depletion in MCF7, PC3 and to a lesser extent HeLa cells (Supplementary Fig. 7a).

A shorter isoform of the CHK1 protein (termed CHK1-S) has previously been reported to be translated from an alternative downstream translational initiation site in exon 3-skipped *CHEK1* mRNA^37^. In our experiments, although depletion of Tra2 proteins switched splicing of *CHEK1* exon 3, they did not lead to an observable increase in any shorter isoform of the CHK1 protein. We detected much lower expression levels of possible shorter CHK1 protein isoforms on western blots compared with full-length CHK1 protein (the ~43 KDa protein that would correspond in size to CHK1-S could only be seen on long exposure, and decreased on siRNA treatment,Supplementary Fig. 7b). CHK1-S protein is reported to be regulated over the cell cycle and in tumours^37^. To test for cell cycle regulated splicing inclusion of *CHEK1* exon 3, we prepared RNAs from KG1 cell populations enriched in different cell cycle stages prepared using elutriation (Supplementary Fig. 5). When analysed by RT–PCR, very similar patterns of *CHEK1* exon 3 splicing inclusion were observed in each of the cell populations even though they contain different cell cycle profiles. We could detect high levels of both *CHEK1* splice isoforms in RNA purified from a small panel of breast cancer tissues, although we did not see an enrichment of either isoform in any particular tumour type at the RNA level (Supplementary Fig. 6). Overall, the above data are most consistent with Tra2α and Tra2β activity being essential for expression of full-length CHK1 protein rather than inducing expression of a shorter protein isoform of CHK1.

### Tra2 protein depletion affects DNA damage and cell viability

Although it is not as a direct target of CHK1 phosphorylation, γH2AX has been used as a marker for the replication stress that can be induced by depleted CHK1 levels^38^,^39^. Similar to previous observations^38^, we observed greatly increased levels of the DNA damage marker γH2AX following depletion of CHK1 protein by siRNA, compared with cells treated with a negative control siRNA (Fig. 8a). Increased γH2AX levels were also observed after joint depletion of Tra2α and Tra2β proteins in MDA-MB-231 cells and in MCF7 cells (Fig. 8a,b). The relative increased levels of γH2AX following CHEK1 or TRA2A/B siRNA treatment appear proportional to the reduction in full-length CHK1 protein expression observed by western blot.

Microscopy and MTT assays also indicated reduced cell viability 120 hours after joint Tra2α and Tra2β depletion (Fig. 8c,d). In contrast, single depletion of either Tra2α or Tra2β had negligible effect on cell viability compared with mock depleted cells. Similar results were obtained using two independent sets of siRNAs targeted at different regions of the mRNAs. This reduction in cell viability from joint removal of Tra2α and Tra2β, compared with the negligible effects of removing either protein alone, suggest that Tra2α and Tra2β are functionally interchangeable for maintaining cell viability in MDA-MB-231 cells, as well as in splicing control.

Depletion of CHK1 also reduced cell viability in MDA-MB-231 cells (Fig. 8e). This suggests that depletion of full-length CHK1 protein would likely be sufficient by itself to contribute to the loss of cell viability observed after joint Tra2α and Tra2β depletion. To test if re-introduction of full-length CHK1 protein would be sufficient to restore viability of joint Tra2α and Tra2β protein-depleted cells, we made a stable cell line in the FLP-in HEK-293 cell background in which a full-length FLAG-tagged CHK1 protein was expressed under control of a tetracycline promoter. Similar to the result obtained in MDA-MB-231 cells, joint depletion of Tra2α and Tra2β reduced cell viability in this stable HEK-293 cell line. However, although the full-length FLAG-tagged CHK1 protein was efficiently induced by tetracycline, it was not sufficient to rescue cell viability after joint Tra2 protein depletion (Supplementary Figs 8 and 9). While we cannot rule out that the tagged full-length CHK1 protein failed to rescue viability of this cell line for another reason, this result is consistent with multiple exons controlled by Tra2 proteins (including *CHEK1* exon 3) being important for cell viability.

Finally, we monitored incorporation of the thymidine analogue EdU using flow cytometry to determine whether joint Tra2 protein depletion affected cell proliferation of MDA-MB-231 cells (Fig. 8f). After joint Tra2 protein depletion, we observed a significant reduction in the proportion of EdU-positive cells 96 h after siRNA transfection (an 8.4% reduction, *P*=0.02), indicating fewer cells had initiated DNA replication after joint Tra2 protein depletion. A slight reduction in the proportion of EdU-positive cells was observed after single CHK1 protein depletion, but this was not statistically significant when compared with negative control siRNA-treated cells. Joint Tra2 protein depletion also caused an increase in the proportion of cells containing abnormally shaped nuclei 96 h after siRNA transfection, consistent with major biological defects (Fig. 8g).

## Discussion

Here we find that only joint depletion of both Tra2α and Tra2β proteins (and not single depletion of either protein alone) could induce substantial splicing switches in endogenous Tra2β target exons in MDA-MB-231 cells. This joint depletion strategy has enabled us to derive the most comprehensive map of dual Tra2-dependent target exons in any organism to date. Among the jointly regulated exons identified here was a key exon in the *CHEK1* gene, which encodes a protein essential for monitoring DNA damage and controlling cell cycle progression^37^,^39^. Exon 3 of the *CHEK1* gene is 224 nucleotides long; hence, skipping of this exon in the absence of Tra2 proteins would frameshift the reading frame of the *CHEK1* mRNA if a downstream translational initiation site is not selected^37^. Joint depletion of both Tra2 proteins quantitatively switched *CHEK1* pre-mRNA splicing, reduced expression of full-length CHK1 protein, and led to an increase in DNA damage as monitored by accumulation of γH2AX. We also confirmed *SMN2* exon 7 as a joint Tra2α/Tra2β target exon. Joint control by Tra2α provides an explanation why *SMN2* exon 7 is a target for Tra2β in transfected cells, but not appreciably affected in *Tra2b* single knockout mice^15^.

This strategy also reveals that Tra2 proteins are required for splicing inclusion of some constitutively spliced exons. To the best of our knowledge, Tra2 proteins have only previously been described as alternative splicing factors^26^. Low levels of apparent alternative splicing of constitutive exons might be ascribed to error prone exon recognition by the spliceosome^40^. However, the *ANKRD1* and *SMC4* exons are not annotated as alternatively spliced in any tissue consistent with them being true constitutive exons, yet also show high-amplitude splicing changes upon Tra2 protein depletion. A role in constitutive splicing brings the Tra2 proteins closer to the core SR group in described molecular functions^23^. Consistent with this newly discovered role, Tra2β protein is fairly evenly expressed across mouse tissues, so would be available in most cells for splicing inclusion of constitutive exons^10^,^18^. Although we only detected exons activated by Tra2 proteins in this study, exons have previously been described that are repressed by Tra2 proteins^29^,^41^. Such repressed exons might have either eluded our search criteria or occur less frequently.

Genes containing Tra2-dependent exons are enriched in processes associated with chromosome biology. Although GO annotations are known to be incomplete and can differ in accuracy^42^, this pattern of functional enrichment observed in our data is notably coherent. A potential association between the Tra2-dependent exons and chromosome biology is additionally supported by the connectivity of the BioGRID functional interaction network. In addition to the regulated *CHEK1* alternative splice, 6/17 of the strongest Tra2 protein-responsive exons (showing >40 point PSI change after Tra2 depletion) were also in genes involved in chromosome structure and epigenetic regulation, including *MSL3* (−41 point PSI exon switch after Tra2 depletion). *MSL3* is the human ortholog^43^ of the *Drosophila melanogaster MSL3* gene, which regulates chromatin remodelling during sex determination, and is under control of Sex Lethal (a protein just upstream of Tra2 in the *Drosophila* sex determination pathway) in flies. The *SMC4* gene (−66 point PSI exon switch after Tra2 depletion) encodes a protein important for DNA repair and chromosome condensation, and also interacts with the CTCF transcription factor that modifies chromatin structure^44^. The *ANKRD1* (−73 point PSI exon switch after Tra2 depletion) encodes a transcription factor, which is a negative regulator of cardiac genes^45^. Also among the genes with highly Tra2 protein-responsive exons was *GLYR1* (−41 point PSI exon switch after Tra2 depletion), which is a cofactor for histone demethylation^46^, and the zinc-finger protein *ZCCHC9* (−68 point PSI exon switch after Tra2 depletion), which targets histone mRNAs for degradation^47^. *ZCCHC7* (−72 point PSI exon switch after Tra2 depletion) and *ZCCHC11* (−46 point PSI exon switch after Tra2 depletion) encode zinc-finger proteins homologous to ZCCHC9, but with roles in non-coding RNA metabolism^47^,^48^,^49^. The *MPHOSPH10* gene (−42 point PSI exon switch after Tra2 depletion) encodes a protein involved in ribosomal RNA processing in interphase, and is associated with chromosomes during mitosis^50^. Other genes involved in chromatin modification that are controlled by Tra2 proteins but did not fit into the most responsive group include the *NASP* (*NASP-T*, −12 point PSI exon switch after Tra2 depletion) and *ATRX* genes (−23 point PSI exon switch after Tra2 depletion)^18^,^51^. Interestingly, the two known *Drosophila* Tra2 splicing targets *Doublesex* and *Fruitless* are both transcription factors^12^, and one of the major functions of the *Drosophila* sex determination pathway is dosage compensation via chromatin modification. Our data lend further support to the association of particular splicing regulators with the regulation of coherent cellular functions, also described for NOVA, RBFox2, PTB and T-STAR^4^,^5^,^6^,^7^.

Our data indicate a high degree of functional redundancy between Tra2α and Tra2β, and a powerful homeostatic repressive feedback activity of Tra2β over Tra2α that buffers splicing changes when just one of these proteins is missing. Although splicing defects after single Tra2α and Tra2β depletion were small, they were often individually statistically significant (for example, in the *ATRX* gene). Such fine tuning of splicing profiles by joint Tra2 protein concentration by splicing feedback control might be important in whole organisms and at particular points of development (for example, in brain or testis development). Even individually, small splicing defects over many Tra2β-target exons might cumulatively cause physiological defects. This might explain why *Tra2b*-null mice are embryonic lethal despite containing a *Tra2a* gene^15^,^17^,^18^. Similar asymmetric expression patterns, in which a dominantly expressed splicing factor cross-regulates other family member proteins, have been found in the PTB family, where PTBP1 cross-regulates PTBP2 and PTBP3 (ref. 53). Comprehensive identification of PTBP1 targets similarly required joint depletion of PTBP1 and PTBP2 (ref. 53) Future studies of Tra2β-regulated splicing may also benefit by considering expression levels of both Tra2 proteins, rather than in the context of Tra2β expression alone.

Joint depletion of both Tra2 protein levels reduced cell viability in MDA-MB-231 cells, likely at least in part because of the requirement for productive splicing of the *CHEK1* mRNA. CHK1 protein expression is critical to reduce replication stress in cancer cells undergoing rapid proliferation driven by oncogenes including *RAS* and *MYC*^54^,^55^. Our data thus suggest the Tra2 proteins may represent novel targets to inhibit cancer cell growth.

## Methods

### Cell culture

MDA-MB-231 cells and MCF7 cells were maintained in DMEM (no phenol red) plus 10% fetal bovine serum and 1% Penicillin Streptomycin. HEK-293, HeLa and PC3 cells were maintained in DMEM plus 10% fetal bovine serum. Cells lines were originally purchased from the American Type Culture Collection and LGC Standards, Europe.

### iCLIP

Triplicate iCLIP experiments were performed following the iCLIP protocol^33^. Briefly, MDA-MB-231 cells were irradiated with 400 mJ cm^−2^ ultraviolet-C light on ice, lysed and subject to partial RNase digestion. The crosslinked Tra2β-RNA complexes were then immunoprecipitated using Protein A Dynabeads (Invitrogen) and a rabbit polyclonal anti-Tra2β antibody (Abcam, ab31353). cDNA libraries were prepared according to the published iCLIP protocol. High throughput sequencing of cDNA libraries was performed using an Illumina GAIIx.

### RNA-seq

RNA was extracted from cells using RNeasy Plus Mini Kit (Qiagen) following manufacturer’s instructions and re-suspended in nuclease-free water. All RNA samples were DNase treated using DNA-free kit (Invitrogen) and stored at −80 °C prior to RNA quality control check using 2100 Agilent Bioanalyser and mRNA library prep using TruSeq mRNA library kit (Illumina). Pair-end sequencing was done in total for six samples (three biological replicates of negative control siRNA-treated cells and three biological replicates from *TRA2A* and *TRA2B* siRNA-treated cells) using an Illumina HiSeq 2000.

### Bioinformatics (iCLIP and RNA-seq analysis)

iCLIP data analysis, crosslink site identification and quantification, randomization of iCLIP positions and pentamer enrichment analysis were performed according to published procedures^33^. Briefly, we used the human genome annotation version hg19, and gene annotations from Ensembl 59. Experiment barcode and random barcodes were registered and removed from iCLIP reads. After trimming, we ignored reads shorter than 11 nucleotides. Remaining trimmed reads were then mapped using Bowtie^56^, allowing two mismatches and accepting only reads with single hits. Crosslink sites were initially identified as the first nucleotide upstream of the iCLIP tag, and then filtered to determine statistically significant crosslink sites and those which occurred in clusters within 15 nucleotides windows and with a significant iCLIP tag count, compared with randomized positions, as described in Konig *et al*.^56^. For RNA-seq analysis, the base quality of raw sequencing reads were checked with FastQC (ref. 57) and refined with Seqtk (ref. 58) and Trim-galore (ref. 59). Reads were mapped to the hg19 reference with Tophat2 (ref. 60) and matches analyzed with Bedtools (ref. 61). Differentially expressed genes and exon usage were determined with DESeq (ref. 62) and DEXSeq (ref. 63) respecitvely.

### siRNA transfection

Efficient knockdown of endogenous Tra2α, Tra2β and CHK1 proteins were achieved by transfecting cells with Silencer Select Pre-designed siRNAs (Ambion), targeting *TRA2A* mRNA (Ambion IDs: s26664 and s26665), *TRA2B* mRNA (Ambion IDs: s12749 and s12751) or *CHEK1* mRNA (Ambion ID: s503) respectively, with siPORT NeoFX Transfection Agent (Ambion). Control cells were transfected with a negative control siRNA (Ambion Cat#: 4390843). MDA-MB-231 cells grown in 100 mm tissue culture dishes were transfected with either 24 μl of 10 μM negative control siRNA (control), 12 μl of 10 μM siRNA targeting *TRA2A, TRA2B or CHEK1* (single Tra2α, Tra2β or CHK1 knockdown) or 12 μl of 10 μM siRNA targeting *TRA2A* and 12 μl of 10 μM siRNA targeting *TRA2B* (joint Tra2α and Tra2β knockdown). Cells were incubated for 72 h post siRNA transfection, before RNA extraction or western blotting.

### Splicing assays: RNA extraction, RT–PCR and PCR

RNA was extracted using standard Trizol RNA extraction. cDNA was synthesized from 500 ng total RNA in a 10 μl reaction, using Superscript VILO cDNA synthesis kit (Invitrogen) following manufacturer’s instructions. Splicing profiles were monitored by PCR using primers in flanking exons. For each PCR, 1 μl diluted cDNA (1/8) was used as template in a 10 μl PCR reaction using Phusion High-Fidelity PCR Kit (NEB, UK) following manufacturer’s instructions. Splicing profiles were monitored and quantified using the Qiaxcel capillary electrophoresis system (Qiagen) and PSI was calculated as described previously^18^. All primers used for splicing assays are provided in Supplementary Data 3.

### Quantitative PCR

Relative gene expression was determined by quantitative real-time PCR using the SYBR Green PCR Master Mix kit (Applied Biosystems) and an Applied Biosystems 7900HT Fast Real-Time PCR Machine. cDNA was generated from equal quantities of total RNA for each sample using Superscript VILO cDNA synthesis kit (Invitrogen) following manufacturer’s instructions. Gene expression was calculated relative to three housekeeping genes *ACTB*, *GAPDH* and *TUBB*. *C*_*t*_ values for each sample were calculated using SDS 2.4 software (Applied Biosystems) and relative mRNA expression was calculated using the 2^**−**ΔΔ*Ct*^ method.

### Calculation of Tra2β binding site density

Tra2β binding site density was calculated as the percentage of nucleotides within an exon that correspond to the top 10 kmers identified from the Tra2β iCLIP experiments (Supplementary Fig. 2b).

### Detection of proteins using western blotting

Endogenous proteins were detected by western blot analysis using the following primary antibodies and dilutions: Tra2α (Novus Biologicals, H00029896-B01P;1:500 dilution), Tra2β (Abcam, ab31353;1:2,000 dilution), CHEK1 (Proteintech, 10362-1-AP;1:250 dilution), Histone H2AX (Santa Cruz Biotechnology, sc54-606;1:500 dilution), γH2AX (Ser 139) (Santa Cruz Biotechnology, sc-101696;1:500 dilution), FLAG (Sigma-Aldrich, F3040;1:2,000 dilution), β-Actin (Sigma-Aldrich, A5441,1:2,000 dilution) and α-Tubulin (Sigma-Aldrich, T5168;1:2,000 dilution).

### *ANKRD1 and CHEK1* minigene construction and mutagenesis

The *ANKRD1* constitutive exon and ~200 nucleotides of flanking intronic region was amplified from human genomic DNA using the cloning primers ANKRD1 F (5′- AAAAAAAAAGAATTCAAAATCTAAGACTTGCTTATGGCATT -3′) and ANKRD1 R (5′ AAAAAAAAAGAATTCAGCATGAGAGTTACCGTGAGC -3′). The PCR products were digested with *BamH1* restriction enzyme and cloned into the pXJ41 vector^64^ using the *Mfe*1 site midway through the 757 nucleotide rabbit β-globin intron 2. Tra2β binding site mutations were made using site directed mutagenesis with the following primers; ANKRD1 M1F (5′- AGAACACATATCAAAGCTTGCACATTTATACGACCTTGAAA -3′), ANKRD1 M1R (5′- CAAGGTCGTATAAATGTGCAAGCTTTGATATGTGTTCTAG -3′), ANKRD1 M2F (5′- ATCATTCAACTGCAGCAACGGCAACAATACAGGCACACTAAAG -3′) and ANKRD1 M2R (5′- GAACTTTAGTGTGCCTGTATTGTTGCCGTTGCTGCAGTTGAATG -3′). The *CHEK1* alternative exon and approximately 250 nucleotides of flanking intronic region was synthesised *in vitro* and similarly cloned into the pXJ41 vector. A mutated version that disrupted Tra2β binding sites was also synthesised (sequence provided in Fig. 7c) and cloned into the pXJ41 vector. Analysis of splicing patterns of mRNAs transcribed from minigenes was carried out in HEK-293 cells as previously described^18^,^24^, using primers within the β-globin exons of pXJ41; PXJRTF (5′- GCTCCGGATCGATCCTGAGAACT -3′) and PXJB (5′- GCTGCAATAAACAAGTTCTGCT -3′).

### EMSAs

Gel shift experiments^18^,^65^ were performed using full-length Tra2β protein and *in vitro*-translated RNA probes made from constructs containing amplified regions of the human *CHEK1* gene, cloned into the pBluescript vector. Three regions of the human *CHEK1* gene were amplified using the following primers: CHEK1 intronic F (5′- AAAAAAAAAGGTACCTGTGTACCTCTCCTTCACTACC -3′), CHEK1 intronic R (5′- AAAAAAAAAGAATTCCTGTCCTAAGCTCCTATGGGG -3′), CHEK1 exon region A F (5′- AAAAAAAAAGGTACCgttcaacttgctgtgaatagagt -3′), CHEK1 exon region A R (5′- AAAAAAAAAGAATTCggcacgCTTCAtatctacaATCT -3′), CHEK1 exon region B F (5′- AAAAAAAAAGGTACCagtaaaattctatggtcacagga -3′) and CHEK1 exon region B R (5′- AAAAAAAAAGAATTCctccactacagtactccagaaat -3′).

### GO and functional network analysis

GO^66^ enrichment analysis was carried out using the Bioconductor GOstats package version 2.24.0 refs 67,68. Enrichments of GO biological process terms were calculated using the conditional hypergeometric test with a significance cut-off of 0.001 and using a background of genes that are normally expressed in MDA-MB-231 cells. Annotations were taken from the Bioconductor *Homo sapiens* annotation package org.Hs.eg.db version 2.8.0 ref. 69. The analysis was run in the open source statistical package R version 3.0.1 ref. 70.

Interaction data for *Homo sapiens* was retrieved from the BioGRID database (version 110). These data were integrated into a network in which nodes represented genes or gene products, and edges represented any type of BioGRID interaction between the nodes. The network was visualised using the Cytoscape visualization platform^71^, and was coloured based on annotations to top five enriched GO biological processes (as downloaded from QuickGO^71^). Where a protein was annotated to more than one term, the most specific annotation was chosen.

### MTT assay

MTT assays were performed using MTT Cell Proliferation Assay Kit (Cayman Chemical), following manufacturer’s instructions. An siRNA transfection mix was added to a suspension of ~2 × 10^5^ MDA-MB-231 cells in 10 ml media. The siRNA/cell suspension was gently mixed and a 100 μl aliquot was added per well to a 96-well plate. Absorbance from the MTT assay was measured at 24, 48, 72, 96 and 120 h after siRNA transfection/seeding of cells. Relative density of cells was also compared 120 h after seeding cells by microscopy.

### Fluorescence-activated cell sorting analysis of EdU-positive cells

MDA-MB-231 cells were incubated with 10 μM EdU for 4 h, 96 h after siRNA transfection. Cell fixation, permeabilization and EdU detection was performed using the Click-iT EdU Flow Cytometry Assay Kit (Life Technologies) following the manufacturer’s instructions. Data were collected and analysed using a BD LSR II flow cytometer using 488 nm excitation and a 520/20 band-pass for detection of EdU Alexa Fluor488 azide and 355 nm excitation and a 450/50 band-pass for detection of 4',6-diamidino-2-phenylindole. Experiments were performed with biological triplicate samples and 30,000 cells were analysed per sample. A no-EdU control sample was used to inform our gating strategy to calculate the proportion of EdU-positive cells.

### Analysis of nuclear morphology

To investigate nuclear morphology, cells were fixed with 4% paraformaldehyde followed by nuclear staining with 4',6-diamidino-2-phenylindole, 96 h after siRNA transfection (siRNA transfection as described above).

### Elutriation and cell cycle evaluation

Elutriation: Cells were size fractionated by centrifugal elutriation, using flow rates of 10, 13, 17, 20, 24 and 28 ml min^−1^ (ref. 72). Cell cycle evaluation: Cell cycle phase enrichment of cells was assessed using immunofluorescence staining for CENPF (late S, G_2,_ G_2_/M)^73^ and phospho-histone H3S10 (G_2_/M, M)^74^. Asynchronous and elutriated KG1 cells were suspended in PBS, spotted onto poly-lysine-coated slides and processed for immunofluorescence. Images were captured and cells were scored for CENPF and phospho-H3S10 staining.

### Generation of tetracycline-inducible HEK-293 cells

Full-length CHK1-FLAG cDNA was amplified from the pcDNA4-Chk1-Flag plasmid (Addgene plasmid #22894) using the primers CHEK1 FLAG F (5′- AAAAAAAAAGCGGCCGCatggcagtgccctttgtggaagac -3′) and CHEK1 FLAG R (5′- AAAAAAAAAGTCGACtcatgtggcaggaagccaaatcttc -3′) and cloned into the Flp-In expression vector (pCDNA5). To generate an inducible cell line, the CHK1-FLAG-pCDNA5 vector was cotransfected with the Flp recombinase plasmid (pOG44) into Flp-In HEK-293 cells and selected for using treatment with Hygromycin B. Following Hygromycin B selection, CHK1-FLAG expression was induced by the addition of tetracycline to promote expression CHK1-FLAG expression via a tetracycline-inducible promoter.

## Additional information

**How to cite this article**: Best, A. *et al*. Human Tra2 proteins jointly control a *CHEK1* splicing switch amongst alternative and constitutive target exons. *Nat. Commun.* 5:4760 doi: 10.1038/ncomms5760 (2014).

**Accession codes:** iCLIP and RNA-seq data available via the Gene Expression Omnibus (GEO) using GEO accession number GSE59335.

## Supplementary Material

Supplementary Figures Supplementary Figures 1-15

Supplementary Data 1 iCLIP sequencing read statistics showing number of total reads per replicate, number of cDNAs identied (i.e., reads with unique random barcodes mapped at each position), andnumber of cross-link sites.

Supplementary Data 2 Summary of all 53 Tra2-dependent human exons identified in MDA-MB-231 cellsand 38 exons which had iCLIP coverage but did not respond to joint Tra2a/Tra2β protein depletion.

Supplementary Data 3 Sequences of oligonucleotide primers used in this study.
