# Supplementary material for: Human Tra2 proteins jointly control a CHEK1 splicing switch among alternative and constitutive target exons
Source: Nat Commun. 2014 Sep 11;5:4760. doi: 10.1038/ncomms5760 (PMC4175592; doi:10.1038/ncomms5760)

Supplementary Figure 1.

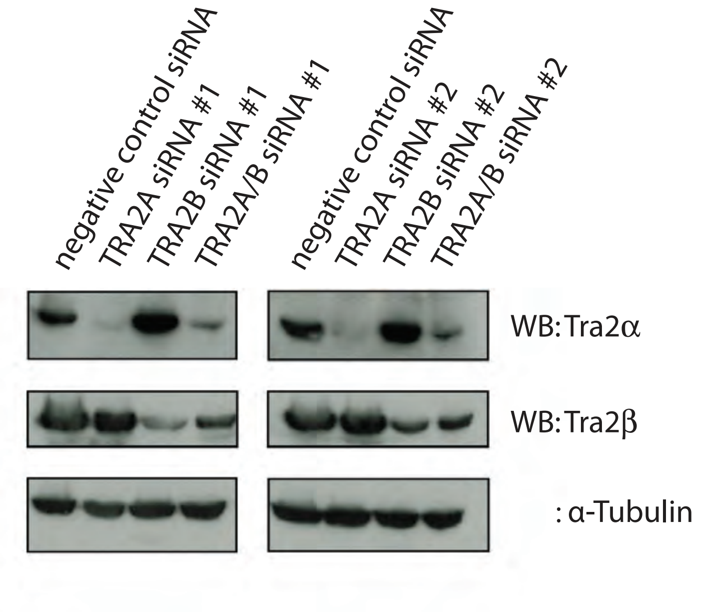

Supplementary Figure 1. Depletion of endogenous Tra2β results in strong up-regulation of endogenous Tra2α protein. Efficient protein knockdown was confirmed by Western blot, using 2 siRNA sequences targeting each of the TRA2A and TRA2B mRNAs. Strong up-regulation of Tra2α was confirmed after independent transfection of MDA-MB-231 cells with two independent siRNAs specific to TRA2B.

Supplementary Figure 2.

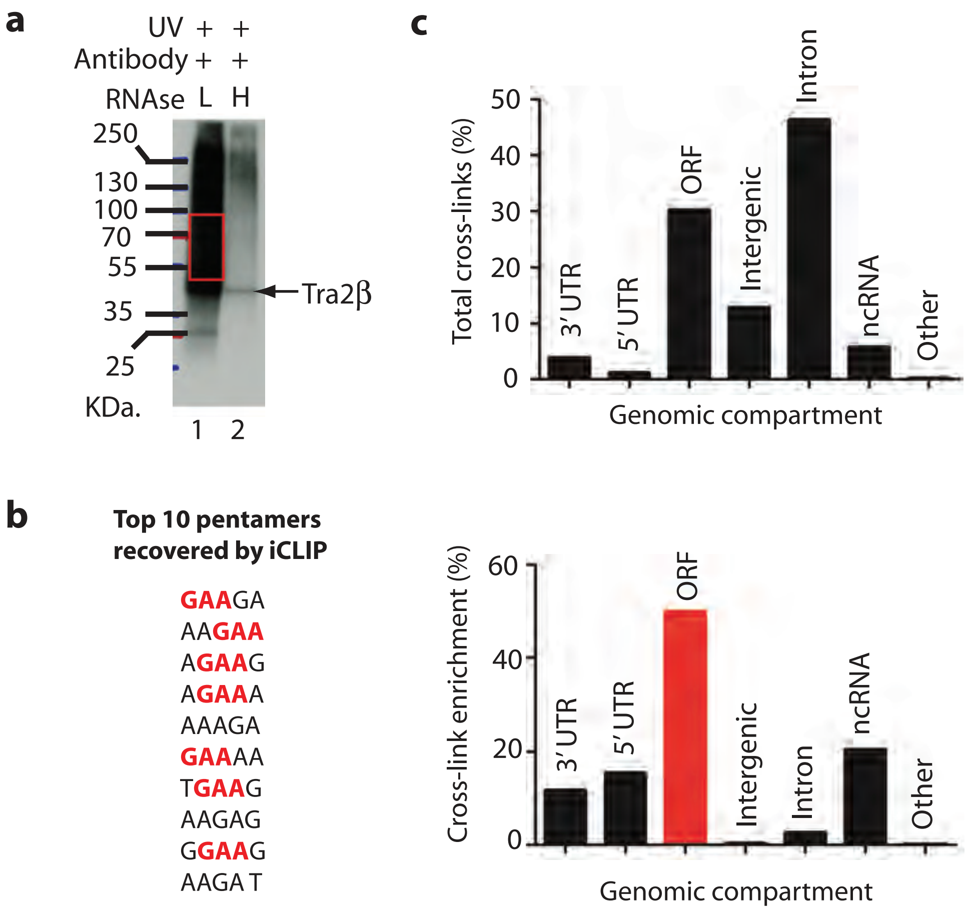

Supplementary Figure 2. Genomic landscape of Tra2β protein binding in MDA-MB-231 cells.
(**a**) Autoradiograph showing 32P-labelled RNA cross-linked to endogenous Tra2β from MDA-MB-231 cells during an iCLIP experiment. The area highlighted in red was excised for further analysis.
(b) The top 10 most frequently occurring pentamers recovered by iCLIP analysis in MDA-MB-231 cells (GAA triplets are highlighted in red).
(c) Genomic distribution of Tra2β binding sites. Total cross-links from triplicate iCLIP experiments (top panel) and relative enrichment of cross-links within each genomic region (number of cross-links relative to the size of each genomic region) (bottom panel).

Supplementary Figure 3.

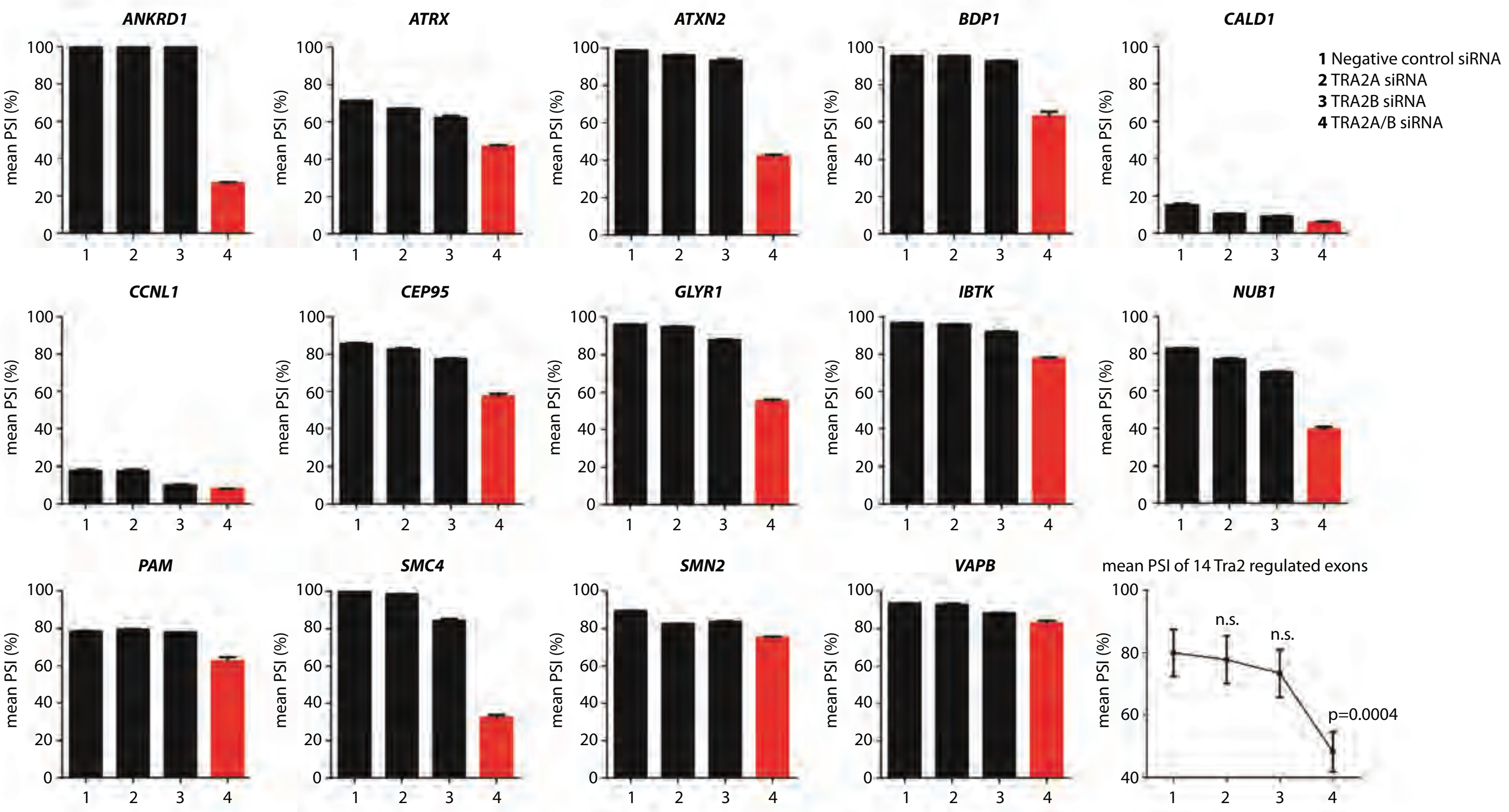

Supplementary Figure 3. RT-PCR validation of 14 Tra2-regulated exons in response to single depletion of Tra2α or Tra2β compared to joint depletion of both Tra2α and Tra2β. Splicing inclusion of 14 Tra2β regulated exons was measured after single depletion of either Tra2α or Tra2β and following joint depletion of both Tra2α and Tra2β simultaneously. PSI levels were measured by RT-PCR using primers in flanking exons in three biological replicates. The mean PSI of all tested Tra2β target exons was not significantly different from the control after single depletion of either Tra2α or Tra2β protein alone, but was highly significant after joint depletion of Tra2α and Tra2β, suggesting robust identification of Tra2-regulated exons would require joint Tra2α and Tra2β depletion prior to RNA-seq analysis.

Supplementary Figure 4.

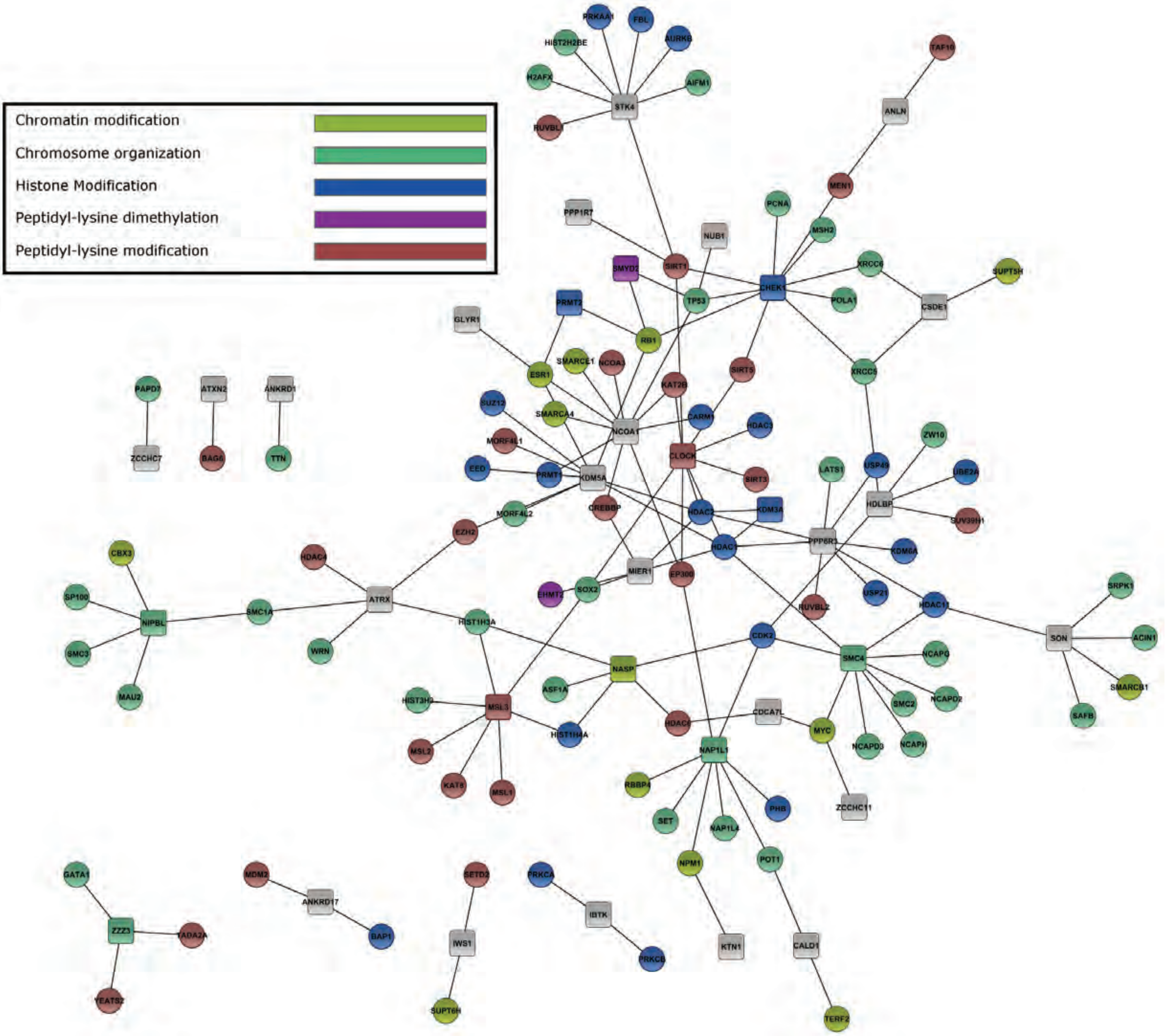

Supplementary Figure 4. Network analysis of Tra2-regulated exons. Network representation of genes whose splicing was functionally responsive to endogenous Tra2 protein (square nodes) and their interactions with genes/proteins annotated to the five enriched GO biological processes (circular nodes). Protein nodes are coloured by GO annotation as downloaded from QuickGO on 27th March 2014 (http://www.ebi.ac.uk/QuickGO/), and edges between nodes represent any type of functional interaction between the genes/proteins present in version 110 of the BioGRID database (http://thebiogrid.org/). Where a protein was annotated to more than one of the terms, the more specific child term was chosen for colouring. However, it should be noted that many of the genes in the network are annotated to multiple enriched terms.

Supplementary Figure 5.

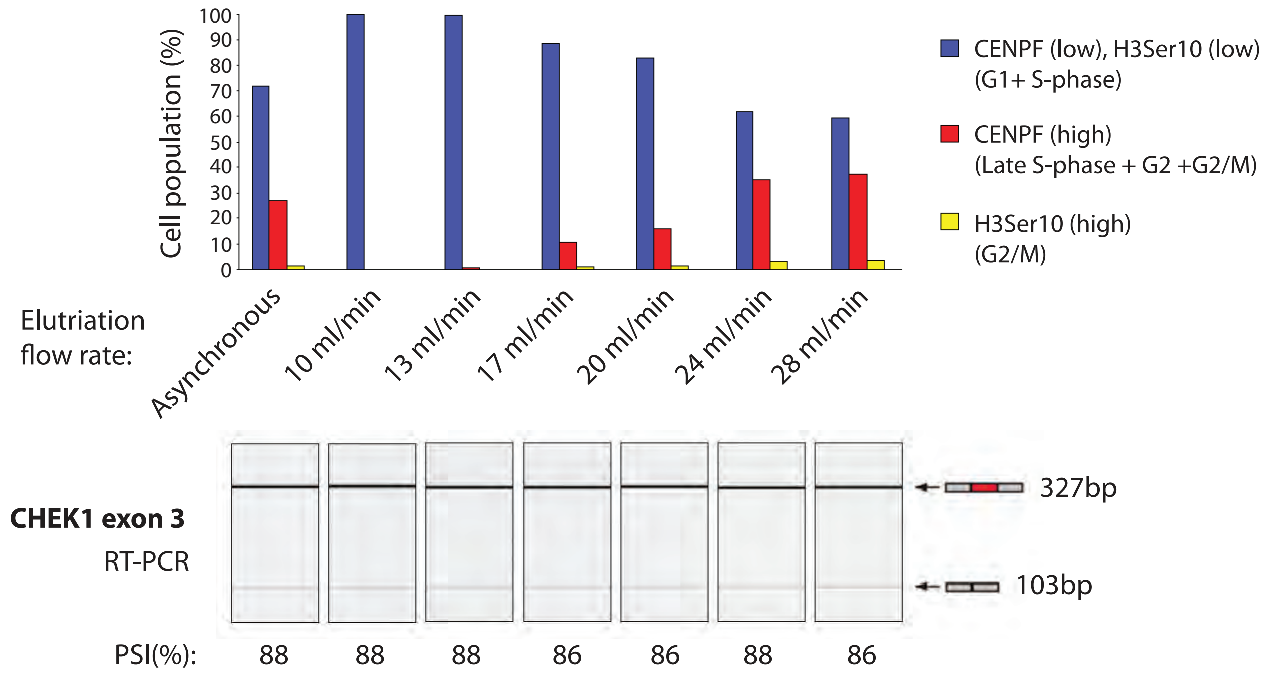

Supplementary Figure 5. (top): Bar chart showing asynchronous KG1 cells and cells from each elutriation flow rate fraction assessed for CENPF expression and phospho-histone H3Ser10 (H3Ser10) by immunofluorescence. For each marker, cells were scored as CENPF high or low and H3Ser10 high or low and the percentage of cells with each pattern plotted. Early fractions are negative for both markers consistent with G1 enrichment. Late S, G2, G2/M -associated CENPF staining appeared in an increasing number of cells from fractions 17-28 ml/min. (Bottom) Capillary gel electrophoretogram showing CHEK1 exon 3 splicing profile in different cell cycle fractions.

Supplementary Figure 6.

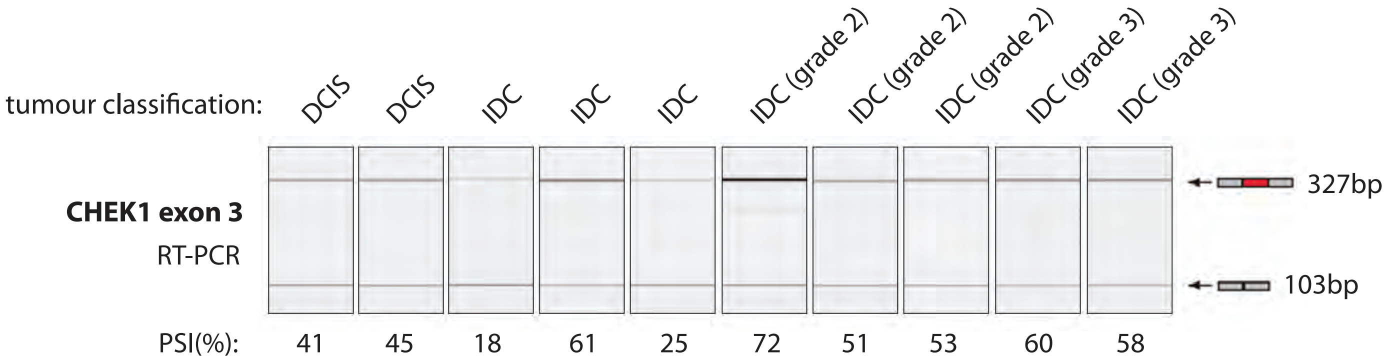

Supplementary Figure 6. CHEK1 exon 3 splicing profile in RNA purified from biopsies of different human breast cancers.

Supplementary Figure 7a.

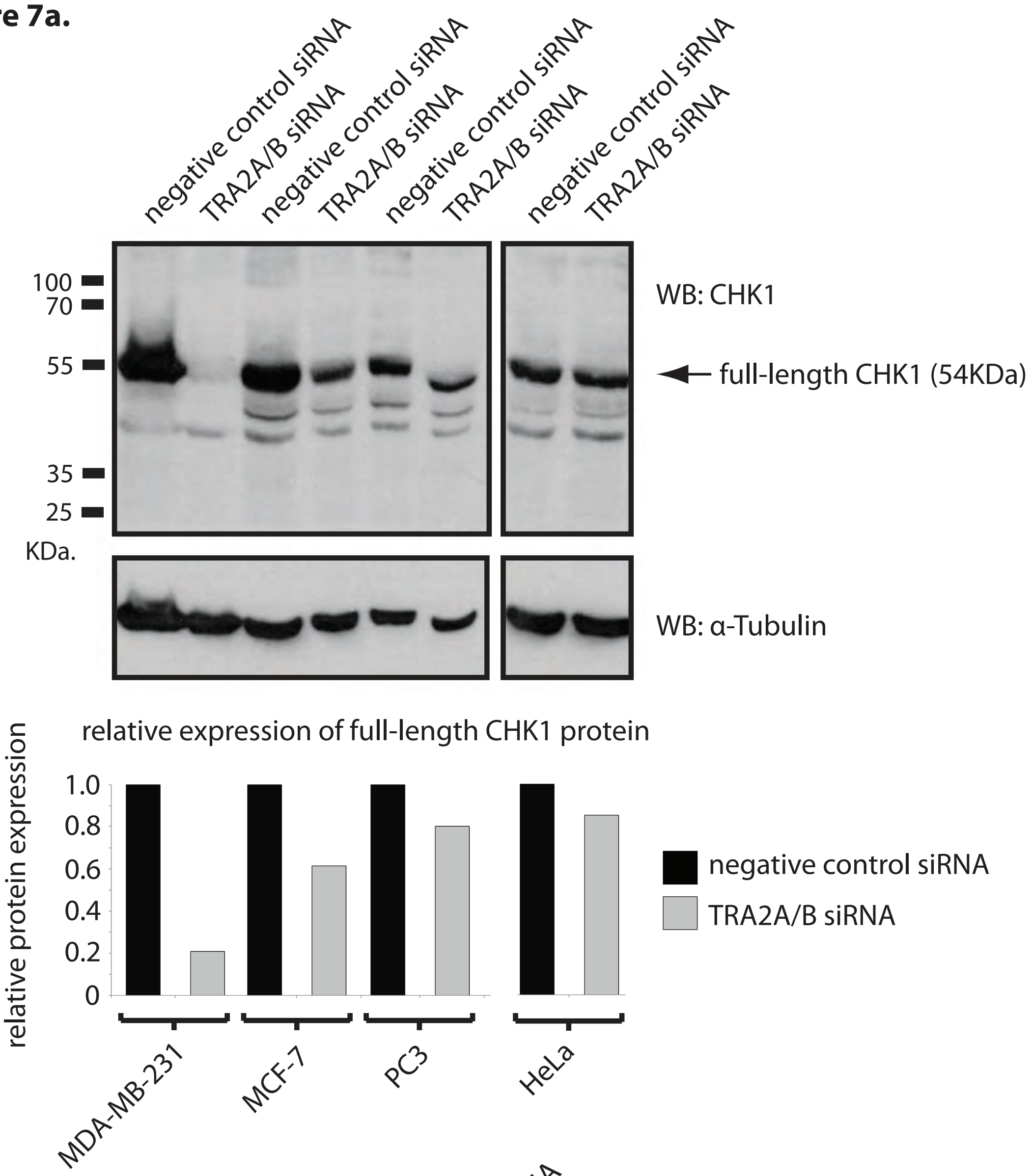

Supplementary Figure 7b.

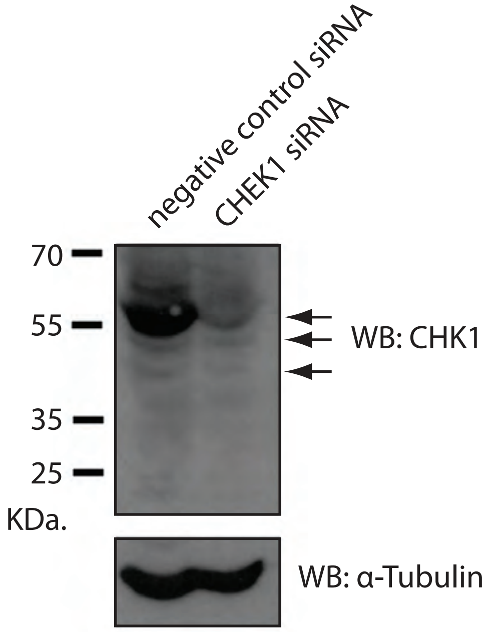

Supplementary Figure 7. (a) Detection of CHK1 protein expression levels by Western blotting (upper panel); α-Tubulin protein expression levels in the same samples by Western blotting (middle panel); and the quantified relative levels of full-length CHK1 protein expression (54kDa band) in cells transfected with negative control siRNA compared to cells transfected with TRA2A/B siRNAs. The cell lines analysed were MDA-MB-231, MCF7, PC3 and HeLa.
(b) Detection of protein isoforms in MDA-MB-231 cells after probing for CHK1 using the Proteintech antibody 10362-1-AP, and using a long period of chemiluminescent exposure to help detect less abundant proteins. α-tubulin is used as a loading control.

Supplementary Figure 8.

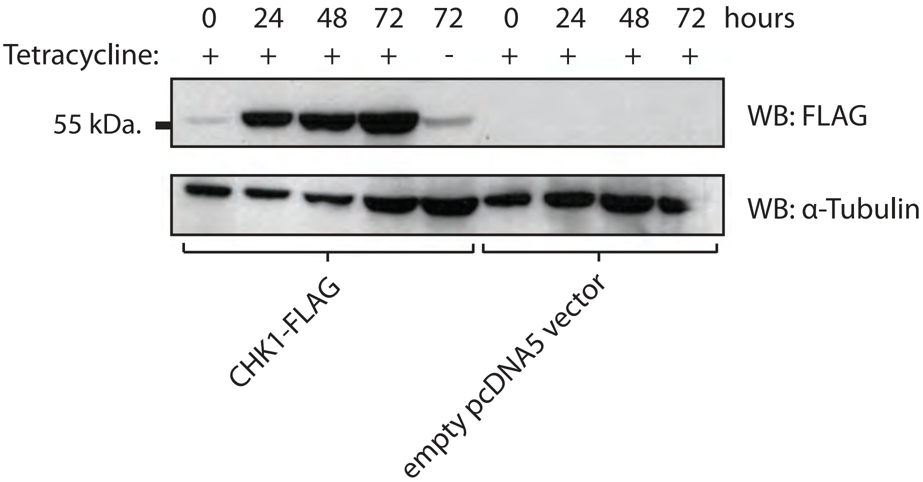

Supplementary Figure 8. Expression of FLAG-tagged CHK1 protein in response to tetracycline induction in a stable FLP-In HEK-293 cell line, compared with a control stable cell line made with empty vector. In each case a time-course of induction is shown after tetracycline addition with α-tubulin detected in parallel as a loading control.

Supplementary Figure 9.

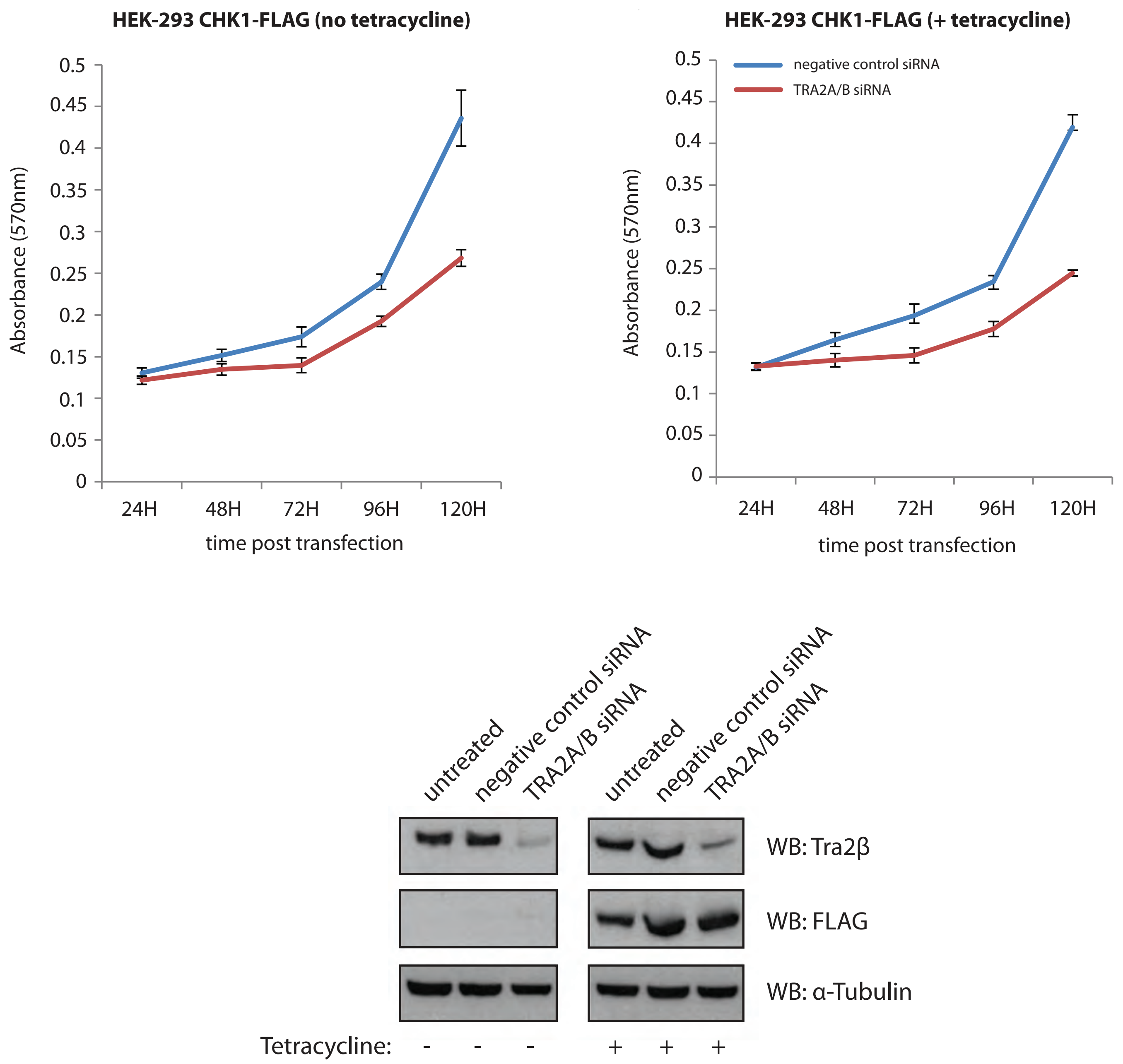

Supplementary Figure 9. Top: Graphs showing cell viability of the stable FLP-In T-REX cells grown without (left graph) or with tetracycline (right graph), following treatment with a control siRNA, or joint siRNA treatment to deplete Tra2α and Tra2β. FLP-In T-REX cell clones were used which stably express either CHK1-FLAG or were created with empty pCDNA5 vector. Underneath: panels showing Western blots from each stable cell line of FLP-In T-REX cells (left hand side, control cells; right hand side, cells expressing CHK1-FLAG) after different siRNA treatments, to show efficiency of Tra2β depletion and expression of CHK1-FLAG protein.

Supplementary Figure 10 - RNA maps of TRA2 responsive exons

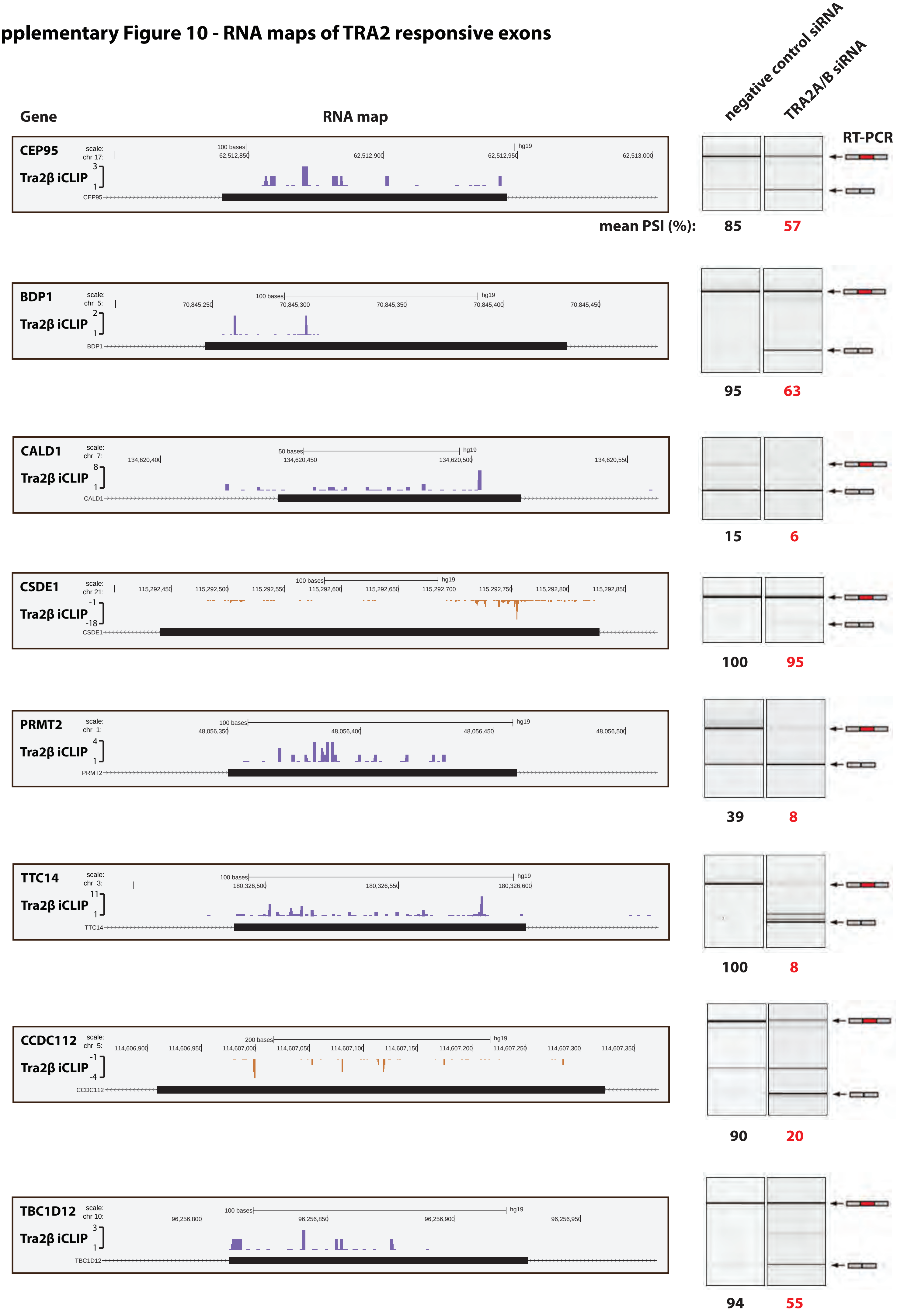

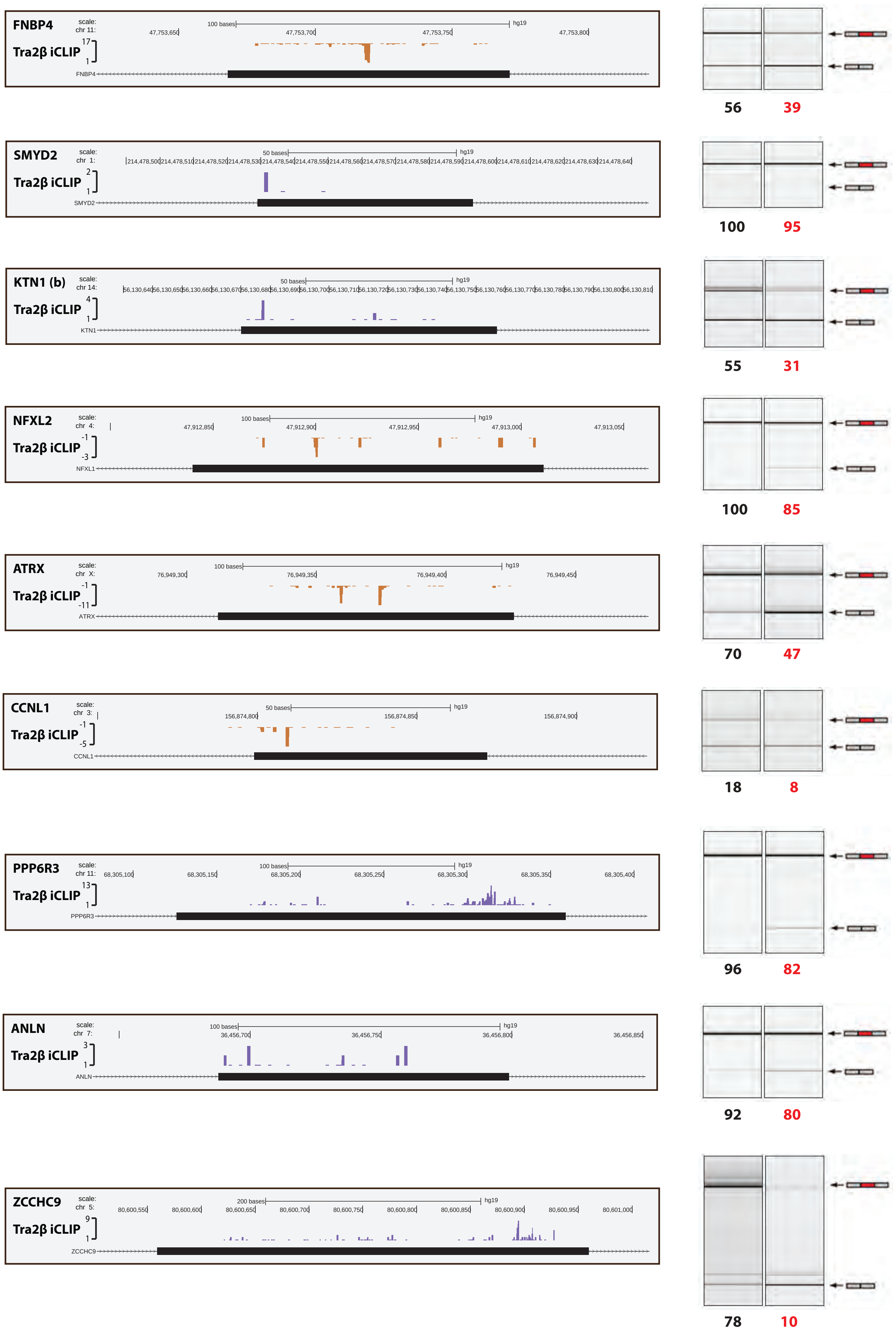

FNBP4
scale:
chr 11:
100 bases
hg19
47,753,650
47,753,700
47,753,750
47,753,800
Tra2β iCLIP
17
1
FNBP4
56
39
SMYD2
scale:
chr 1:
50 bases
hg19
Tra2β iCLIP
2
1
SMYD2
100
95
KTN1 (b)
scale:
chr 14:
50 bases
hg19
Tra2β iCLIP
4
1
KTN1
55
31
NFXL2
scale:
chr 4:
100 bases
hg19
47,912,850
47,912,900
47,912,950
47,913,000
47,913,050
Tra2β iCLIP
-1
-3
NFXL1
100
85
ATRX
scale:
chr X:
100 bases
hg19
76,949,300
76,949,350
76,949,400
76,949,450
Tra2β iCLIP
-1
-11
ATRX
70
47
CCNL1
scale:
chr 3:
50 bases
hg19
156,874,800
156,874,850
156,874,900
Tra2β iCLIP
-1
-5
CCNL1
18
8
PPP6R3
scale:
chr 11:
100 bases
hg19
68,305,100
68,305,150
68,305,200
68,305,250
68,305,300
68,305,350
68,305,400
Tra2β iCLIP
13
1
PPP6R3
96
82
ANLN
scale:
chr 7:
100 bases
hg19
36,456,700
36,456,750
36,456,800
36,456,850
Tra2β iCLIP
3
1
ANLN
92
80
ZCCHC9
scale:
chr 5:
200 bases
hg19
80,600,550
80,600,600
80,600,650
80,600,700
80,600,750
80,600,800
80,600,850
80,600,900
80,600,950
80,601,000
Tra2β iCLIP
9
1
ZCCHC9
78
10

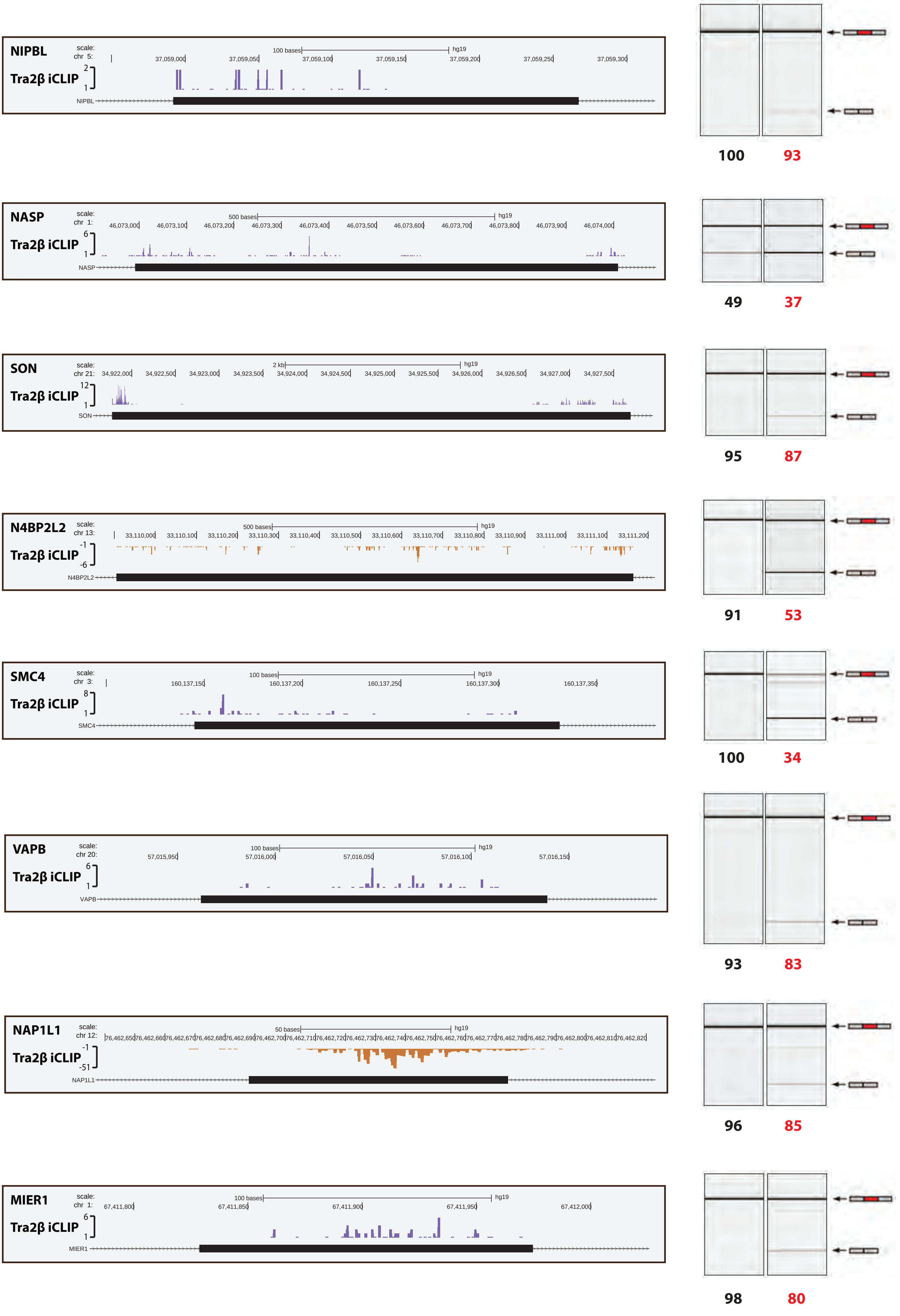

NIPBL
scale:
chr 5:
100 bases
hg19
37,059,000
37,059,050
37,059,100
37,059,150
37,059,200
37,059,250
37,059,300
Tra2β iCLIP
NIPBL
100
93
NASP
scale:
chr 1:
500 bases
hg19
46,073,000
46,073,100
46,073,200
46,073,300
46,073,400
46,073,500
46,073,600
46,073,700
46,073,800
46,073,900
46,074,000
Tra2β iCLIP
NASP
49
37
SON
scale:
chr 21:
2 kb
hg19
34,922,000
34,922,500
34,923,000
34,923,500
34,924,000
34,924,500
34,925,000
34,925,500
34,926,000
34,926,500
34,927,000
34,927,500
Tra2β iCLIP
SON
95
87
N4BP2L2
scale:
chr 13:
500 bases
hg19
33,110,000
33,110,100
33,110,200
33,110,300
33,110,400
33,110,500
33,110,600
33,110,700
33,110,800
33,110,900
33,111,000
33,111,100
33,111,200
Tra2β iCLIP
N4BP2L2
91
53
SMC4
scale:
chr 3:
100 bases
hg19
160,137,150
160,137,200
160,137,250
160,137,300
160,137,350
Tra2β iCLIP
SMC4
100
34
VAPB
scale:
chr 20:
100 bases
hg19
57,015,950
57,016,000
57,016,050
57,016,100
57,016,150
Tra2β iCLIP
VAPB
93
83
NAP1L1
scale:
chr 12:
50 bases
hg19
Tra2β iCLIP
NAP1L1
96
85
MIER1
scale:
chr 1:
100 bases
hg19
67,411,800
67,411,850
67,411,900
67,411,950
67,412,000
Tra2β iCLIP
MIER1
98
80

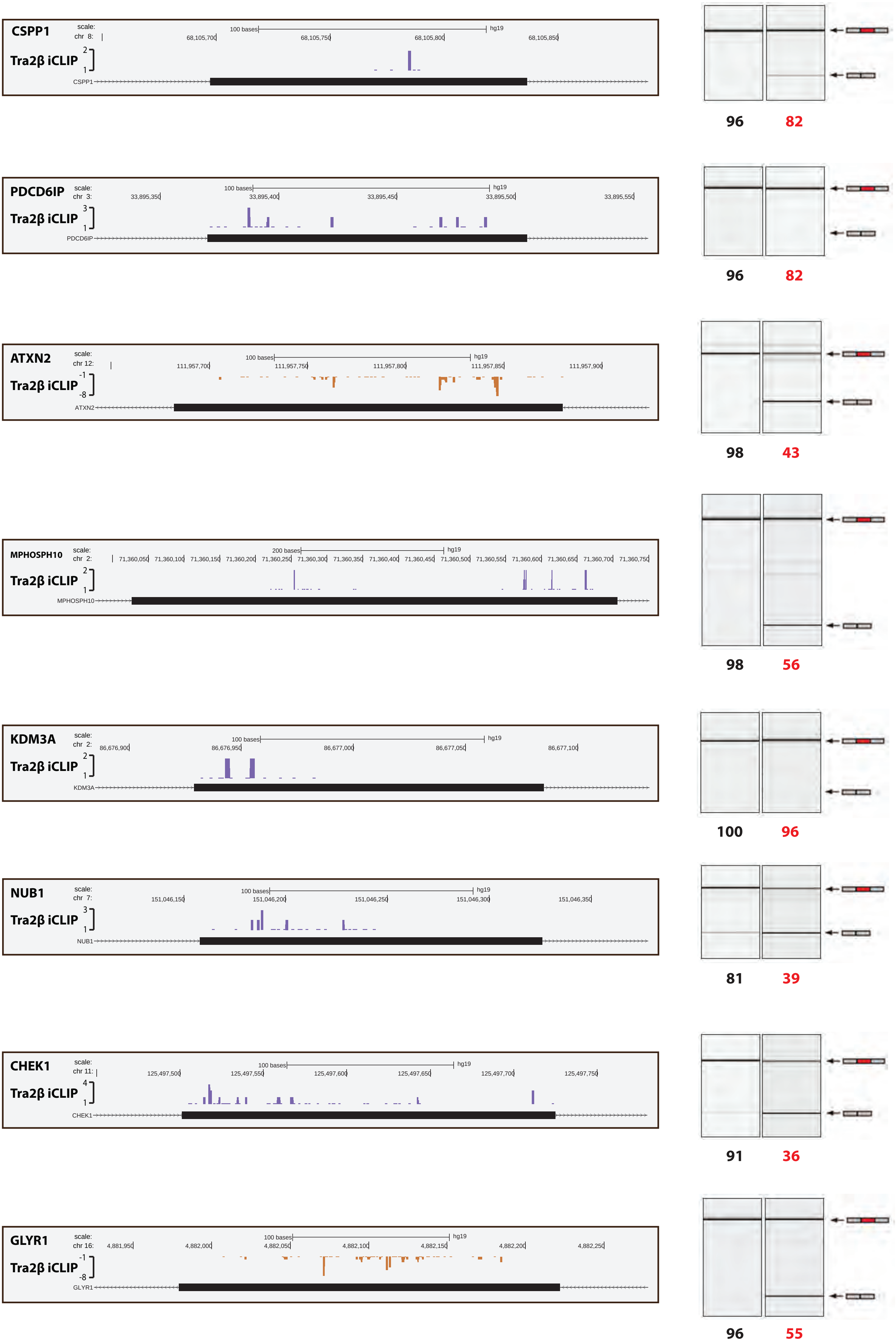

CSPP1
scale:
chr 8:
100 bases
hg19
68,105,700
68,105,750
68,105,800
68,105,850
Tra2β iCLIP
2
1
CSPP1
96
82
PDCD6IP
scale:
chr 3:
100 bases
hg19
33,895,350
33,895,400
33,895,450
33,895,500
33,895,550
Tra2β iCLIP
3
1
PDCD6IP
96
82
ATXN2
scale:
chr 12:
100 bases
hg19
111,957,700
111,957,750
111,957,800
111,957,850
111,957,900
Tra2β iCLIP
-1
-8
ATXN2
98
43
MPHOSPH10
scale:
chr 2:
200 bases
hg19
71,360,050
71,360,100
71,360,150
71,360,200
71,360,250
71,360,300
71,360,350
71,360,400
71,360,450
71,360,500
71,360,550
71,360,600
71,360,650
71,360,700
71,360,750
Tra2β iCLIP
2
1
MPHOSPH10
98
56
KDM3A
scale:
chr 2:
100 bases
hg19
86,676,900
86,676,950
86,677,000
86,677,050
86,677,100
Tra2β iCLIP
2
1
KDM3A
100
96
NUB1
scale:
chr 7:
100 bases
hg19
151,046,150
151,046,200
151,046,250
151,046,300
151,046,350
Tra2β iCLIP
3
1
NUB1
81
39
CHEK1
scale:
chr 11:
100 bases
hg19
125,497,500
125,497,550
125,497,600
125,497,650
125,497,700
125,497,750
Tra2β iCLIP
4
1
CHEK1
91
36
GLYR1
scale:
chr 16:
100 bases
hg19
4,881,950
4,882,000
4,882,050
4,882,100
4,882,150
4,882,200
4,882,250
Tra2β iCLIP
-1
-8
GLYR1
96
55

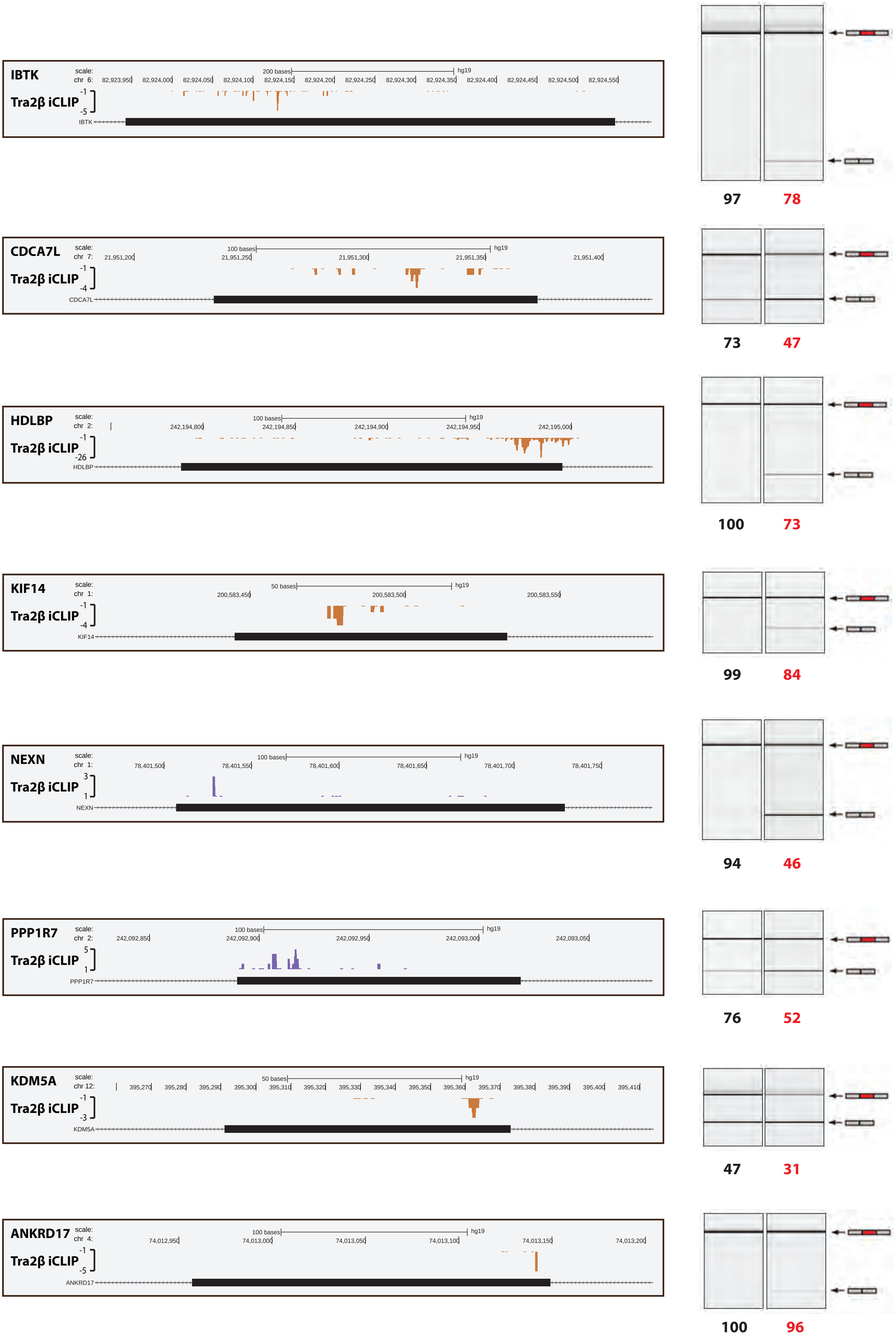
IBTK
scale: chr 6:
200 bases
hg19
82,923,950 82,924,000 82,924,050 82,924,100 82,924,150 82,924,200 82,924,250 82,924,300 82,924,350 82,924,400 82,924,450 82,924,500 82,924,550
Tra2β iCLIP
-1
-5
IBTK
97 78
CDCA7L
scale: chr 7:
100 bases
hg19
21,951,200 21,951,250 21,951,300 21,951,350 21,951,400
Tra2β iCLIP
-1
-4
CDCA7L
73 47
HDLBP
scale: chr 2:
100 bases
hg19
242,194,800 242,194,850 242,194,900 242,194,950 242,195,000
Tra2β iCLIP
-1
-26
HDLBP
100 73
KIF14
scale: chr 1:
50 bases
hg19
200,583,450 200,583,500 200,583,550
Tra2β iCLIP
-1
-4
KIF14
99 84
NEXN
scale: chr 1:
100 bases
hg19
78,401,500 78,401,550 78,401,600 78,401,650 78,401,700 78,401,750
Tra2β iCLIP
3
1
NEXN
94 46
PPP1R7
scale: chr 2:
100 bases
hg19
242,092,850 242,092,900 242,092,950 242,093,000 242,093,050
Tra2β iCLIP
5
1
PPP1R7
76 52
KDM5A
scale: chr 12:
50 bases
hg19
395,270 395,280 395,290 395,300 395,310 395,320 395,330 395,340 395,350 395,360 395,370 395,380 395,390 395,400 395,410
Tra2β iCLIP
-1
-3
KDM5A
47 31
ANKRD17
scale: chr 4:
100 bases
hg19
74,012,950 74,013,000 74,013,050 74,013,100 74,013,150 74,013,200
Tra2β iCLIP
-1
-5
ANKRD17
100 96

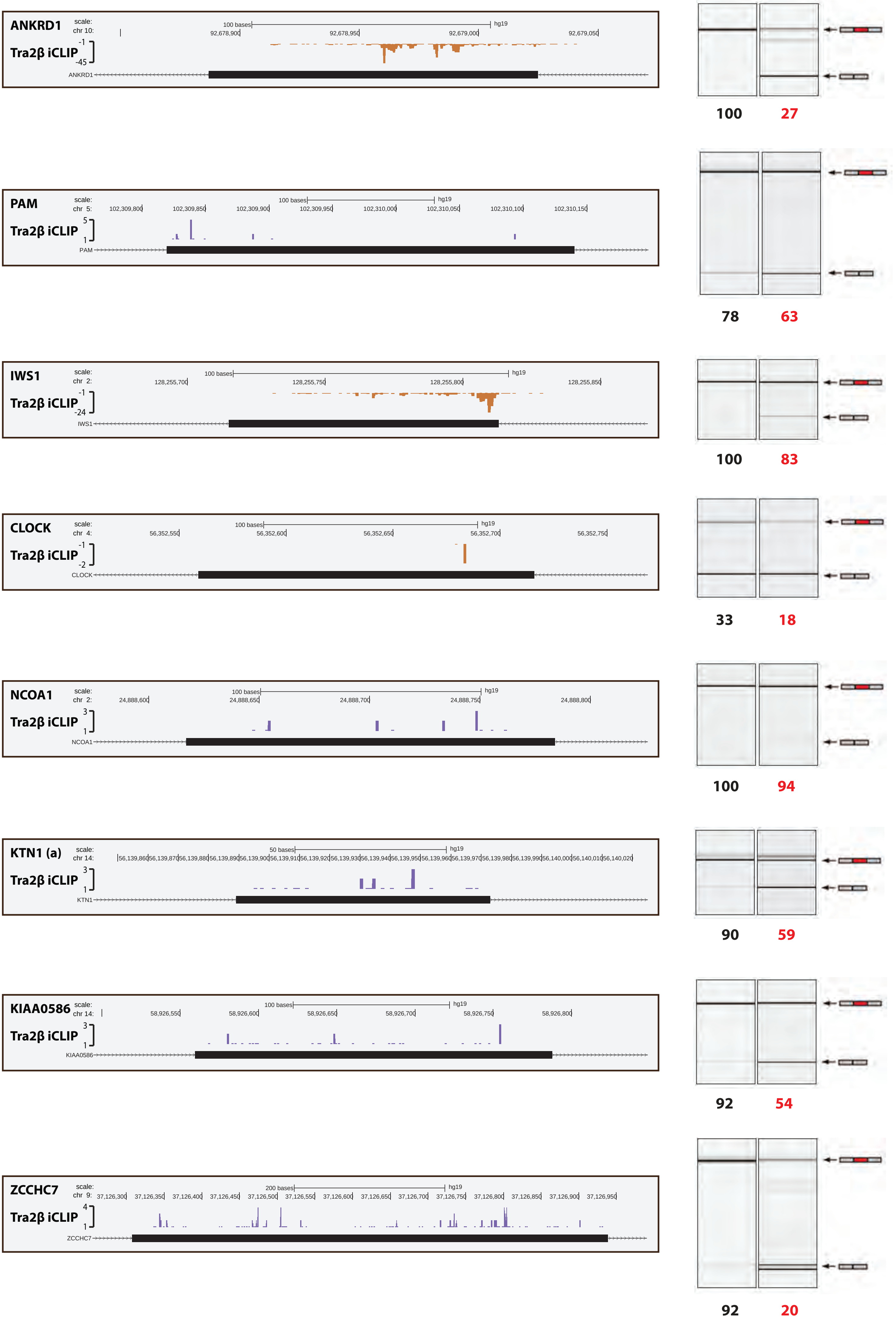

ANKRD1
scale:
chr 10:
100 bases
hg19
92,678,900
92,678,950
92,679,000
92,679,050
Tra2β iCLIP
-1
-45
ANKRD1
100
27
PAM
scale:
chr 5:
100 bases
hg19
102,309,800
102,309,850
102,309,900
102,309,950
102,310,000
102,310,050
102,310,100
102,310,150
Tra2β iCLIP
5
1
PAM
78
63
IWS1
scale:
chr 2:
100 bases
hg19
128,255,700
128,255,750
128,255,800
128,255,850
Tra2β iCLIP
-1
-24
IWS1
100
83
CLOCK
scale:
chr 4:
100 bases
hg19
56,352,550
56,352,600
56,352,650
56,352,700
56,352,750
Tra2β iCLIP
-1
-2
CLOCK
33
18
NCOA1
scale:
chr 2:
100 bases
hg19
24,888,600
24,888,650
24,888,700
24,888,750
24,888,800
Tra2β iCLIP
3
1
NCOA1
100
94
KTN1 (a)
scale:
chr 14:
50 bases
hg19
56,139,860
56,139,870
56,139,880
56,139,890
56,139,900
56,139,910
56,139,920
56,139,930
56,139,940
56,139,950
56,139,960
56,139,970
56,139,980
56,139,990
56,140,000
56,140,010
56,140,020
Tra2β iCLIP
3
1
KTN1
90
59
KIAA0586
scale:
chr 14:
100 bases
hg19
58,926,550
58,926,600
58,926,650
58,926,700
58,926,750
58,926,800
Tra2β iCLIP
3
1
KIAA0586
92
54
ZCCHC7
scale:
chr 9:
200 bases
hg19
37,126,300
37,126,350
37,126,400
37,126,450
37,126,500
37,126,550
37,126,600
37,126,650
37,126,700
37,126,750
37,126,800
37,126,850
37,126,900
37,126,950
Tra2β iCLIP
4
1
ZCCHC7
92
20

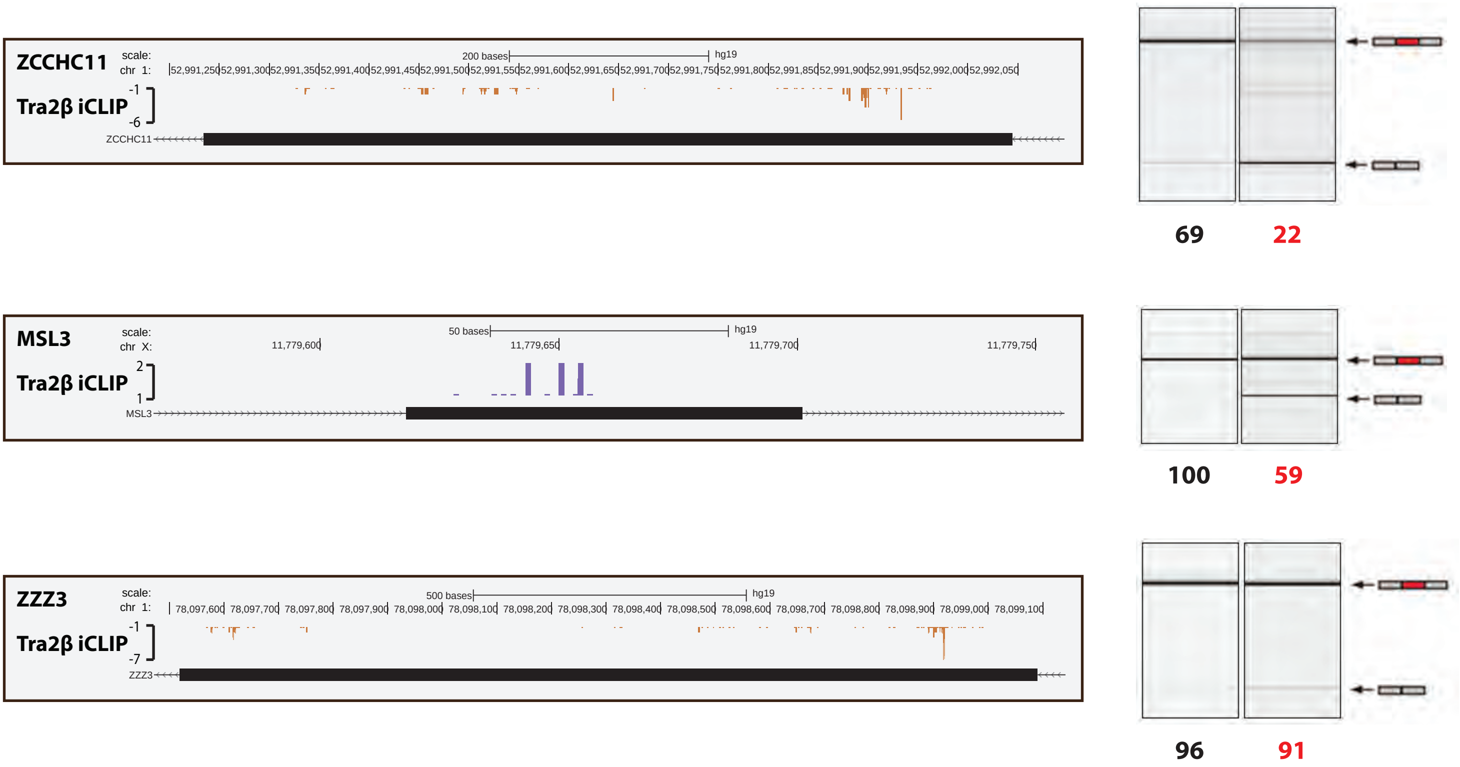

Supplementary Figure 10. iCLIP maps and RT-PCR validation of 53 Tra2-regulated exons that changed splicing profile in response to joint Tra2 depletion. Images of exons that responded to joint Tra2 protein depletion are shown as screenshots from the UCSC genome browser 33. Joint Tra2α/Tra2β responsive exons were identified using combined iCLIP and RNA-seq analysis and splicing was monitored by RT-PCR using primers within flanking exons. PSI (%) levels were measured in MDA-MB-231 cells transfected with a control siRNA or after joint transfection with siRNAs specific to TRA2A and TRA2B in three biological replicates.

Uncropped Western blots and gels

Supplementary Figure 11 - uncropped Western blots corresponding to Figure 1a

WB: Tra2β

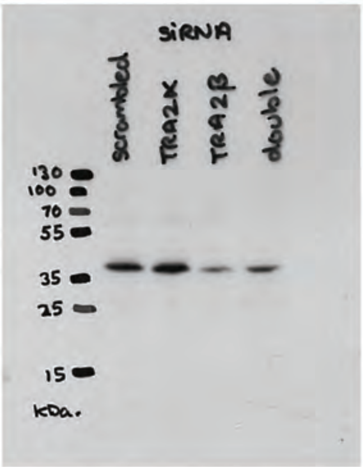

WB: Tra2α

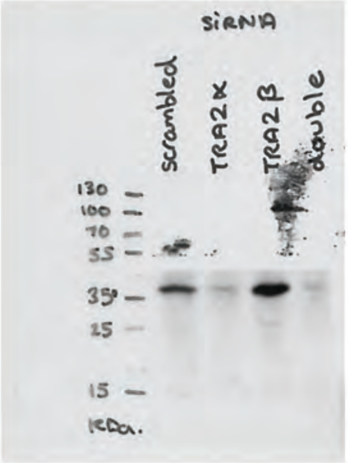

: α-Tubulin

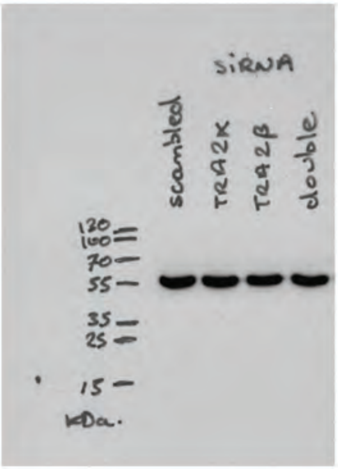

Supplementary Figure 12 - uncropped Western blots corresponding to Supplementary Figure 1

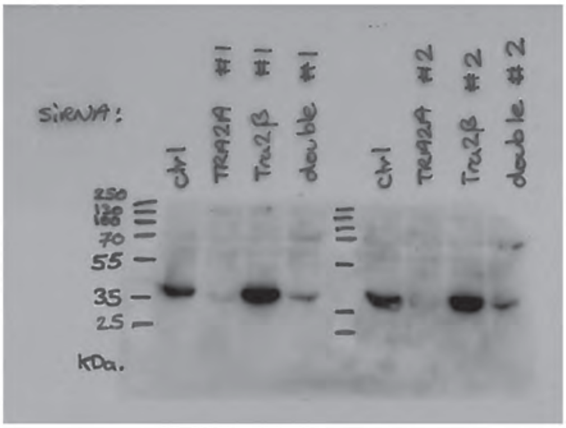

WB: Tra2α

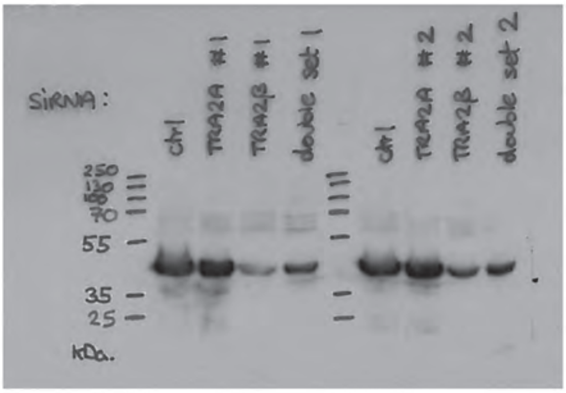

WB: Tra2β

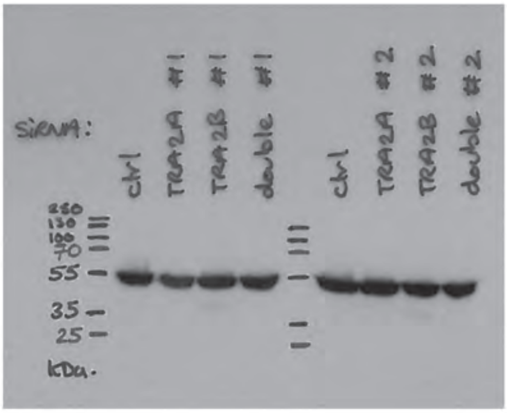

: α-Tubulin

Supplementary Figure 13 - uncropped gel corresponding to Figure 6b

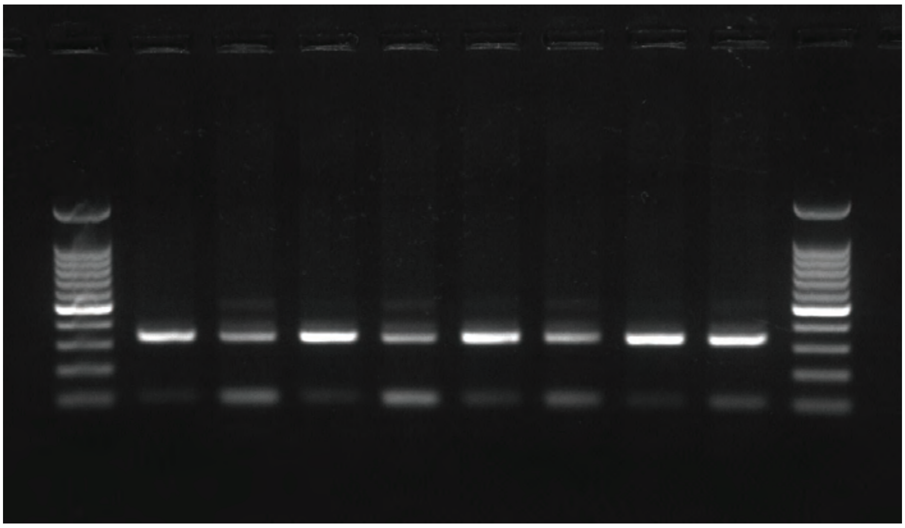

RT-PCR
CHEK1 exon 3

Supplementary Figure 14 - uncropped Western blots corresponding to Figure 8a

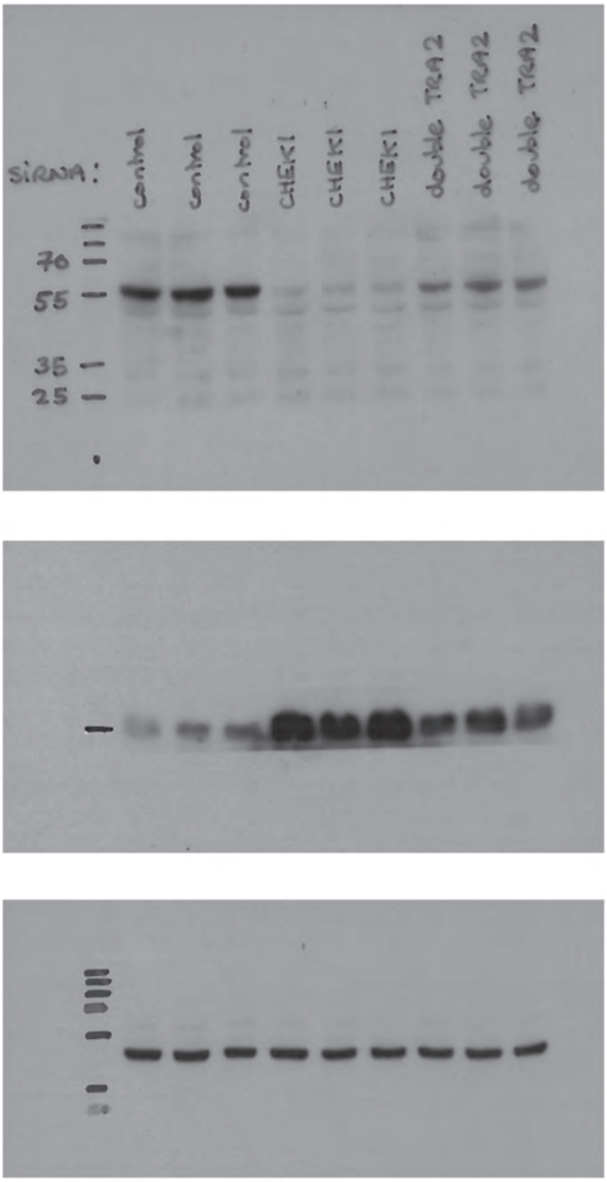

WB: CHK1

WB: γH2AX

: β Actin

Supplementary Figure 15 - uncropped Western blots corresponding to Figure 8b

WB: γH2AX

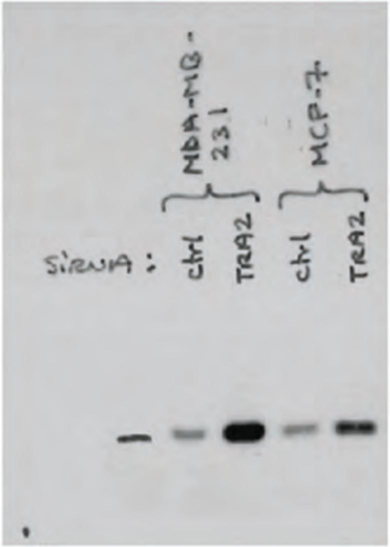

WB: total H2AX

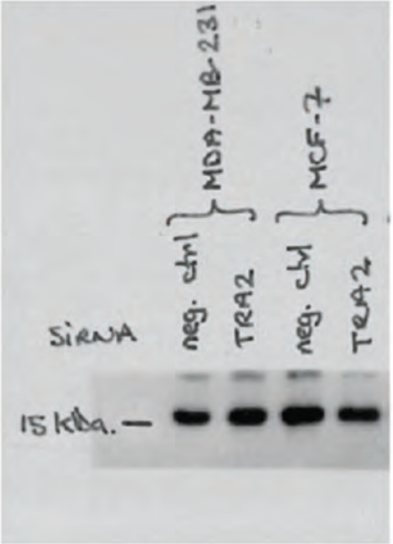

: α-Tubulin

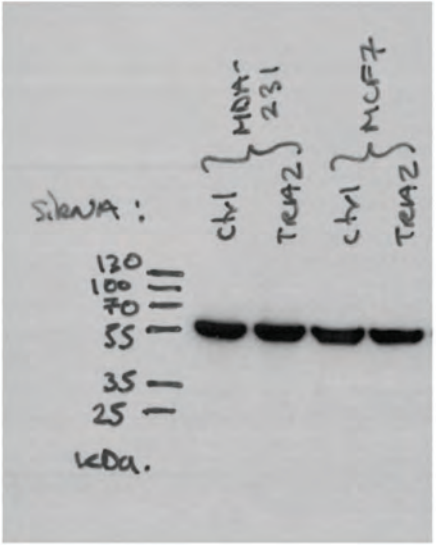

Supplement: Supplementary Figures — 1-15 [file ncomms5760-s1.pdf]
